# Dual‐acceptor engineering of donor‐acceptor type molecules for all‐round boosting anti‐tumor phototherapy

**DOI:** 10.1002/smo.20230014

**Published:** 2023-11-23

**Authors:** Hua Gu, Wen Sun, Jianjun Du, Jiangli Fan, Xiaojun Peng

**Affiliations:** ^1^ State Key Laboratory of Fine Chemicals Frontier Science Center for Smart Materials Dalian University of Technology Dalian China; ^2^ Ningbo Institute of Dalian University of Technology Ningbo China

**Keywords:** anti‐tumor phototherapy, dual‐acceptor engineering, photon‐absorption ability, photothermal conversion efficiency, reactive oxygen yield

## Abstract

The integration of robust photon‐absorption capacity, high reactive oxygen species yields and photothermal conversion efficiency (PCE) into a single phototheranostic nano‐agents is ideal but rarely reported. This study employed a dual‐acceptor engineering strategy utilizing isoindigo and selenium‐substituted [1,2,5]thiadiazolo[3,4‐*c*]pyridine to augment the molar extinction coefficient and spin‐orbit coupling effect, respectively, resulting in a substantial enhancement of photon‐absorption ability and non‐radiative decay energy‐release process of donor‐acceptor type phototherapy molecules. As the optimal phototherapy agent, IID‐PSe exhibited a high molar extinction coefficient two times that of photosensitizer, excellent ^1^O_2_ yield (15%) and PCE (34%), exhibiting great potential for phototherapy. After encapsulating with DSPE‐PEG2000, IID‐PSe NPs showed excellent anti‐tumor phototherapy ability both in vitro and in vivo. This work provides an effective idea for designing high‐performance photosensitive dyes with high efficiency phototherapy output.

## Graphical abstract

We employed a dual‐acceptor engineering strategy to substantially enhance photon‐absorption ability and non‐radiative decay energy‐release process of donor‐acceptor type phototherapy molecules. As the optimal phototherapy agent, IID‐PSe exhibits high molar extinction coefficient, excellent ^1^O_2_ yield and PCE. After encapsulating with DSPE‐PEG2000, IID‐PSe NPs show excellent anti‐tumor phototherapy ability both in vitro and in vivo.

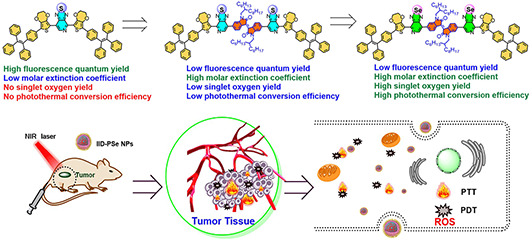

## 1 INTRODUCTION

Phototherapy, which includes photodynamic therapy (PDT) and photothermal therapy (PTT), is recognized as one of the most promising treatment strategies for cancer due to its accuracy and non‐invasibility.[^1^, ^2^, ^3^] For PDT, photosensitizers (PSs) at triplet‐state sensitize oxygen to produce high levels of reactive oxygen species (ROS), kill cancer cells, and achieve tumor suppression through the apoptotic pathway.[^4^, ^5^, ^6^] Therefore, PDT is an oxygen‐depende nt process, and its function is limited due to the anoxic environment of the tumor.[^7^, ^8^] PTT is a typical photothermal conversion therapy strategy. Photosensitizers in the excited state return to the ground state by a non‐radiative transition that releases heat, causing local heat shock that kills tumor cells by apoptotic and/or necrotic pathways without oxygen consumption.[^9^, ^10^] The low concentration and light dose of PS guarantee its good biosafety in PDT.[^11^, ^12^] In contrast, current PTT needs PSs have a strong absorption in the near infrared region and a relatively high concentration and light dose, but are non‐oxygen dependent. Obviously, the advantages and disadvantages of the two treatment methods are complementary.[^13^, ^14^, ^15^] Therefore, PDT combined with PTT is considered a good way to improve the efficacy of tumor therapy.

As an important part of phototherapy materials, donor‐acceptor (D‐A) type organic small‐molecule PSs have attracted much attention due to their easy structure modification, tunable optical properties, excellent stability and high biosafety.[^16^, ^17^, ^18^] But the weak photon‐absorption ability and single phototherapy output of this kind of PSs undoubtedly limit the efficiency of tumor therapy. In our previous work, 2,1,3‐benzothiadiazole‐based fused‐ring strategy with a D‐A structure was used to achieve high molar extinction coefficient and photothermal conversion efficiency (PCE),^[19]^ and single‐molecule Förster resonance energy transfer ‐ based organic PS utilized the radiative transition energy to improve photon utilization.^[20]^ Recently, Tang et al. used a dual‐acceptor strategy to enhance intramolecular D‐A conjugation and intramolecular charge transfer (ICT) effect, which achieved effective regulation of optical properties and PCE.^[21]^ However, the simultaneous improvement of photon utilization, ROS yield and PCE is still rarely reported.

**FIGURE 1:**
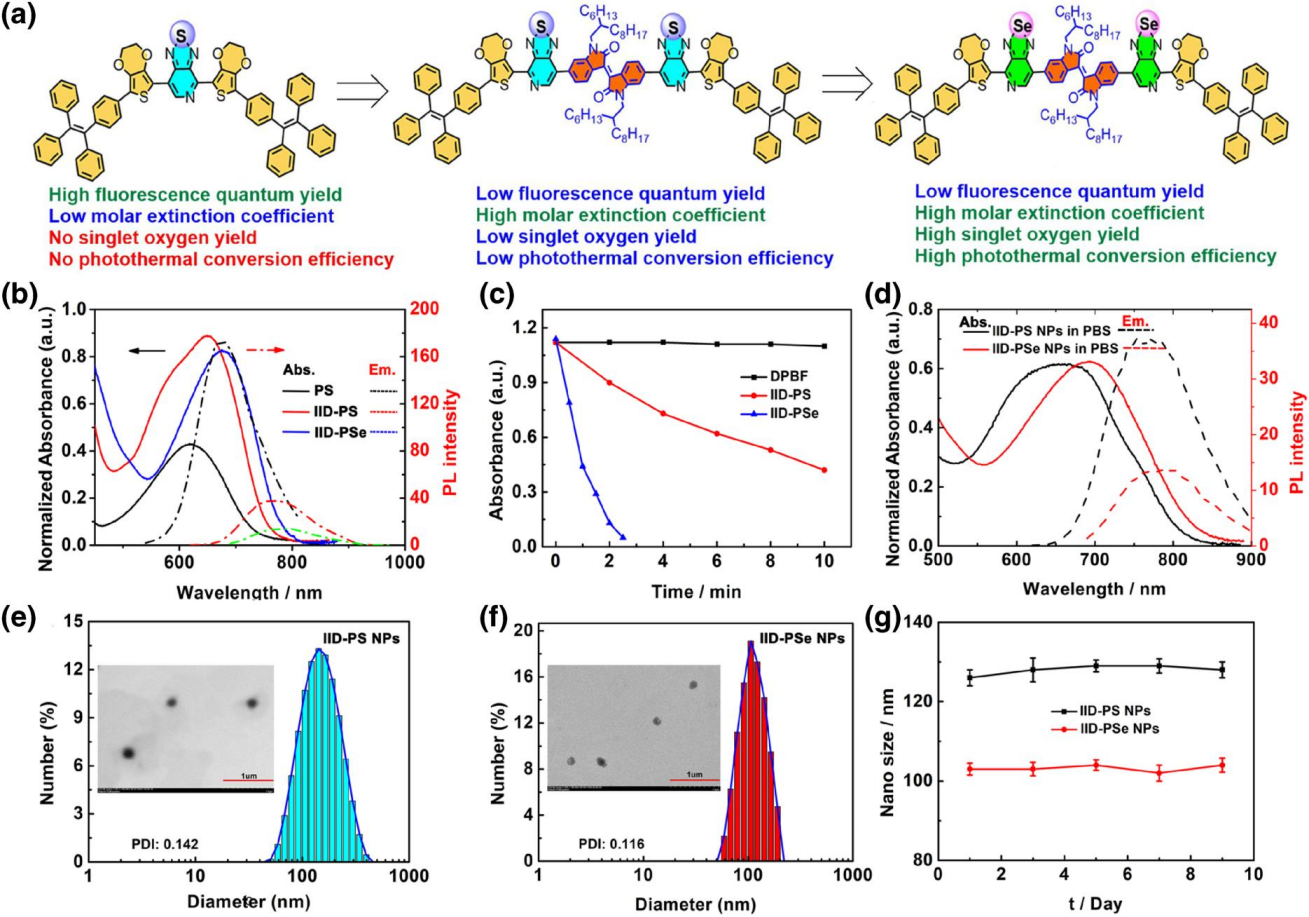
(a) Molecular structures and their multifunctional application. (b) Normalized absorption and photoluminescence (PL) spectra of PS, IID‐PS, and IID‐PSe in CH_2_Cl_2_. (c) Normalized 1,3‐diphenylisobenzofuran (DPBF) absorbance change at 415 nm after being treated with 671 nm irradiation. (d) Normalized absorption and PL spectra of IID‐PS NPs and IID‐PSe NPs in PBS. (e) and (f) DLS and TEM (inset) of IID‐PS NPs and IID‐PSe NPs, respectively. (g) The chemical stability of IID‐PS NPs and IID‐PSe NPs in PBS.

In this study, D‐A type compound PS was synthesized with [1,2,5]thiadiazolo[3,4‐*c*]pyridine as acceptor and 3,4‐ethylenedioxythiophene‐tetraphenylethylene as donor (Figure 1a and Figure S1), which exhibited high fluorescence quantum yield, but low molar extinction coefficient and no photosensitization ability. Isoindigo (IID) structure with large shielding unit and high molar extinction coefficient as the second acceptor was introduced into PS to strengthen the intramolecular D‐A conjugation and ICT effect, which was beneficial to red‐shift absorption wavelength and enhance photon‐absorption capacity.[^22^, ^23^] Furthermore, the alkyl shielding unit of IID prevented intermolecular π‐π accumulation and provided motion space for tetraphenylethylene. As a result, IID‐PS showed certain ^1^O_2_ generation ability and PCE. Selenium (Se) compounds generally have a large atomic radius and dipole moment, which implies a stronger spin‐orbit coupling property and electronegativity than organic sulfur (S) compounds with a similar structure.[^24^, ^25^] Furthermore, Se compounds exhibit weak Se‐Se interaction, which is conducive to improving the intramolecular electron transfer performance of the aggregated state and the PCE.[^26^, ^27^] Therefore, the heavy atom strategy of replacing S atoms with Se atoms was carried out to reduce orbital level, heighten the spin‐orbit coupling effect of IID‐PSe. The final compound and important intermediates were fully characterized (Figures S1–S12). We believe that this design concept of dual‐acceptors will provide new thinking of PSs development for combining anti‐tumor phototherapy.

## 2 RESULTS AND DISCUSSION

As shown in Figure 1b, PS had the maximum absorption at 618 nm with a molar extinction coefficient of 2.8 × 10^4^ M^−1^ cm^−1^ and the maximum emission peak at 645 nm with an absolute fluorescence quantum yield of 14.0%. When introducing the IID structure in IID‐PS, the conjugation length was increased and the ICT effect was enhanced, which contributed to the red‐shift of the absorption and fluorescence spectra to 650 and 765 nm, respectively. As expected, the molar extinction coefficient of IID‐PS was reached to 5.8 × 10^4^ M^−1^ cm^−1^, twice enhanced as the molar extinction coefficient of PS. However, due to the fluorescence quenching effect of additional IID, the absolute fluorescence quantum yield of IID‐PS decreased significantly, lower than 1.4%. After replacing the S atoms in IID‐PS with Se atoms, the optical absorption and emission spectra of IID‐PSe further redshifted to 677 and 775 nm, respectively. Also, its fluorescence property was further weakened with the absolute fluorescence quantum yield of only 0.6%. However, due to the presence of IID, IID‐PSe still maintained a high molar extinction coefficient (5.6 × 10^4^ M^−1^ cm^−1^), which was conducive to enhancing photon‐absorption ability. Furthermore, the ^1^O_2_ generation capacity of IID‐PS and IID‐PSe was assessed through the employment of 1,3‐diphenylisobenzofuran (DPBF). As depicted in Figure 1c and S13, negligible DPBF absorption signal alteration was observed in PBS upon exposure to 671 nm laser irradiation. However, DPBF absorption was reduced by 70% within IID‐PS in just 10 min, and it completely disappeared within IID‐PSe after only 2.5 min, which demonstrated the excellent ^1^O_2_ generation ability of IID‐PSe. This outcome can be attributed to the heavy atom effect resulting from the introduction of Se atoms, which promotes the intersystem crossing (ISC) process and ^1^O_2_ generation ability.[^24^, ^28^] The ^1^O_2_ yield of IID‐PSe was 15%, higher than that of most D‐A type PS dyes.[^29^, ^30^, ^31^]

After wrapping the nanoparticles with DSPE‐PEG2000 (Figure S14), the absorption peaks of IID‐PS NPs and IID‐PSe NPs were widened (Figure 1e) at the maximum absorption at 653 and 694 nm, respectively. As shown in Figures 1e–f, the average hydration particle size (DLS) of IID‐PS NPs and IID‐PSe NPs were concentrated about 126 and 103 nm with the dispersion coefficients of 0.14 and 0.12, respectively, which exhibited a good dispersion and uniformity. In addition, the nanosize of IID‐PS NPs and IID‐PSe NPs in TEM images showed 70–100 nm, which mainly caused by the amphiphilic polymer chain DSPE‐PEG2000 shrinks during the water loss process.^[32]^ Through continuous monitoring for 9 days, the DLS of two NPs remained basically unchanged, proving that the prepared nanoparticles have excellent stability (Figure 1g), which is very important for the biosafety of nanoparticles when they circulate in the body.

**FIGURE 2:**
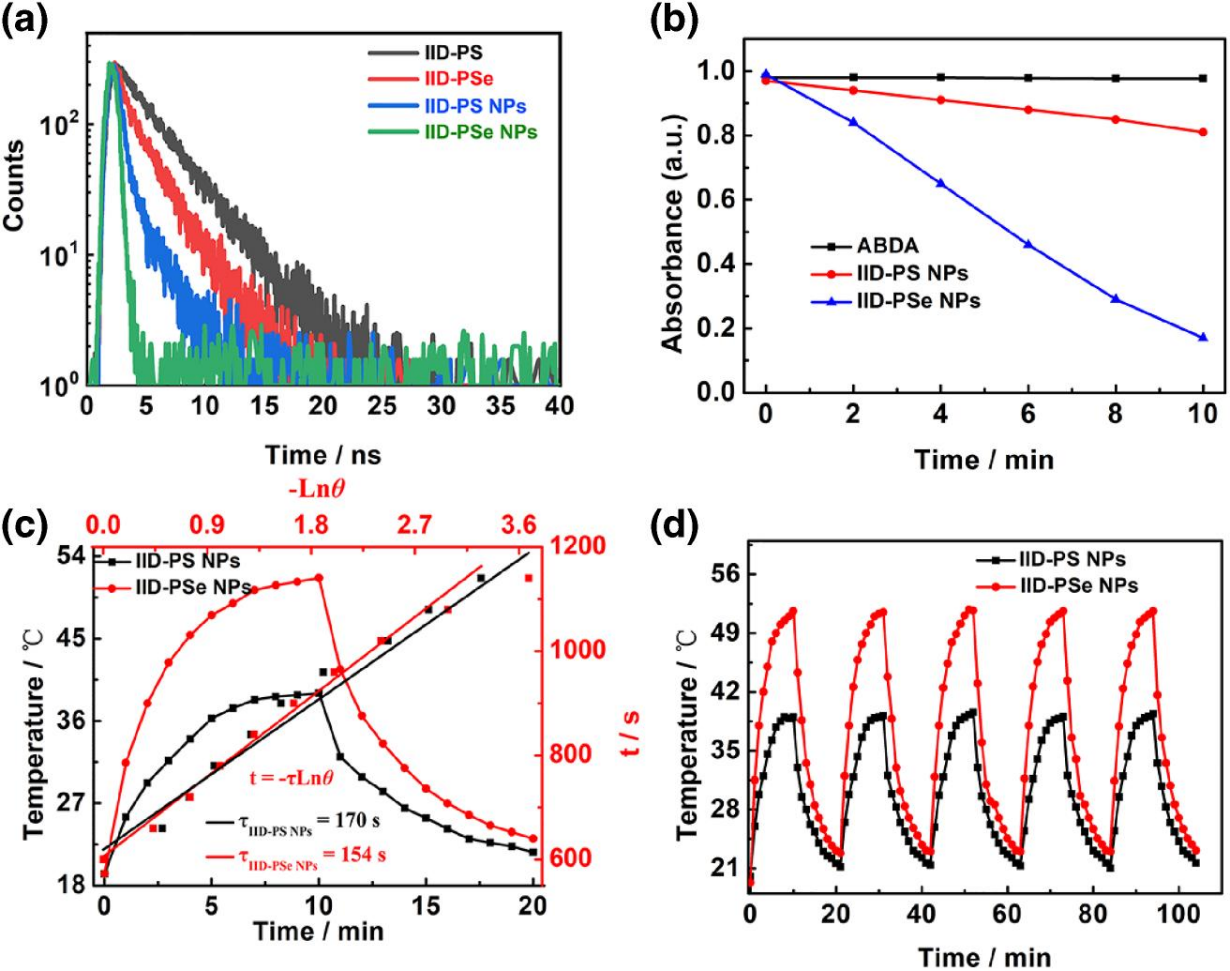
(a) Fluorescence lifetime decay spectra of IID‐PS, IID‐PSe, and their NPs. Excited wavelength: 425 nm; concentration: 25.0 μg mL^−1^. (b) The absorbance change of ABDA with different NPs at 415 nm under 671 nm irradiation and 50.0 mW cm^−2^. (c) Study on photothermal properties of IID‐PS NPs and IID‐PSe NPs under 671 nm laser irradiation (0.5 W cm^−2^). (d) photothermal stability of IID‐PS NPs and IID‐PSe NPs under 671 nm laser irradiation (0.5 W cm^−2^).

Fluorescence lifetime detection was carried out to explore the fluorescence performance. The fluorescence lifetimes of IID‐PS/IID‐PSe in CH_2_Cl_2_ were 3.5 and 2.2 ns respectively after excitation (Figure 2a), which was mainly caused by the spin‐orbit coupling of IID‐PSe itself.[^24^, ^25^, ^28^] When it's wrapped into nanoparticles, the fluorescence lifetime of IID‐PS NPs/IID‐PSe NPs were greatly reduced to 0.4 and 0.1 ns, respectively. It was attributed to aggregation‐induced suppression of radiative dissipative paths.[^33^, ^34^] This result is consistent with those obtained by emission spectra and absolute fluorescence quantum yield. In theory, the reduction of radiation transition will enhance the non‐radiation transition to a certain extent, which is conducive to enhance the effect of phototherapy.[^35^, ^36^]

9,10‐Anthracenediyl‐bis(methylene)dimalonic acid (ABDA) was used as a ^1^O_2_ indicator to demonstrate the production ability of IID‐PS NPs and IID‐PSe NPs in physiological environments. In Figure 2b and S13, when IID‐PS NPs were added, the ABDA absorption slightly showed attenuation after 10 min of laser radiation. However, the participation of IID‐PSe NPs made the absorption spectra of ABDA attenuate rapidly to 20% after 10 min of laser irradiation, which indicated that IID‐PSe NPs could more efficiently produce ^1^O_2_ under the same conditions. It was mainly due to the enhanced spin‐orbit coupling effect and ISC process by heavy atoms of Se.[^25^, ^28^] In addition, with the participation of NPs, the fluorescence spectra of both dihydrorhodamine 123 and hydroxyphenyl fluorescein were not altered (Figure S15), indicating that neither IID‐PSe NPs and IID‐PSe NPs could produce superoxide anion and hydroxyl radical.

Next, the photothermal properties of IID‐PS NPs and IID‐PSe NPs were studied in detail under the same conditions. Firstly, IID‐PS NPs and IID‐PSe NPs showed good photobleaching resistance with the unchanged absorption spectra after 10 min of 671 nm‐laser irradiation (50.0 mW cm^−2^) (Figure S16). The temperature curves of IID‐PS NPs and IID‐PSe NPs at different concentrations were also tested (Figure S17). With the increase in concentration, the temperature of IID‐PS NPs and IID‐PSe NPs gradually increases under the radiation of 671 nm. Similarly, with the increase in the laser power density from 0.1 to 0.5 W cm^−2^, both the temperatures also increased gradually. These results reflected the strong concentration/power‐temperature dependency relationship, which was conducive to flexible temperature control. Compared with PBS under the same laser irradiation, its temperature was basically unchanged, indicating that the temperature increase was mainly attributed to the photothermal conversion ability of nanoparticles. It is worth noting that IID‐PSe NPs always showed higher photothermal performance than that of IID‐PS NPs under the same condition. We attributed it to the stronger spin orbit effect of Se to promote non‐radiative transition decay.^[25]^ Through the rise‐fall temperature curve, we calculated the PCE of 22% for IID‐PS NPs and 35% for IID‐PSe NPs. Besides, IID‐PS NPs and IID‐PSe NPs maintained negligible changes in both photothermal properties and color during five cycles and exhibited good stability and excellent bleaching resistance.

**FIGURE 3:**
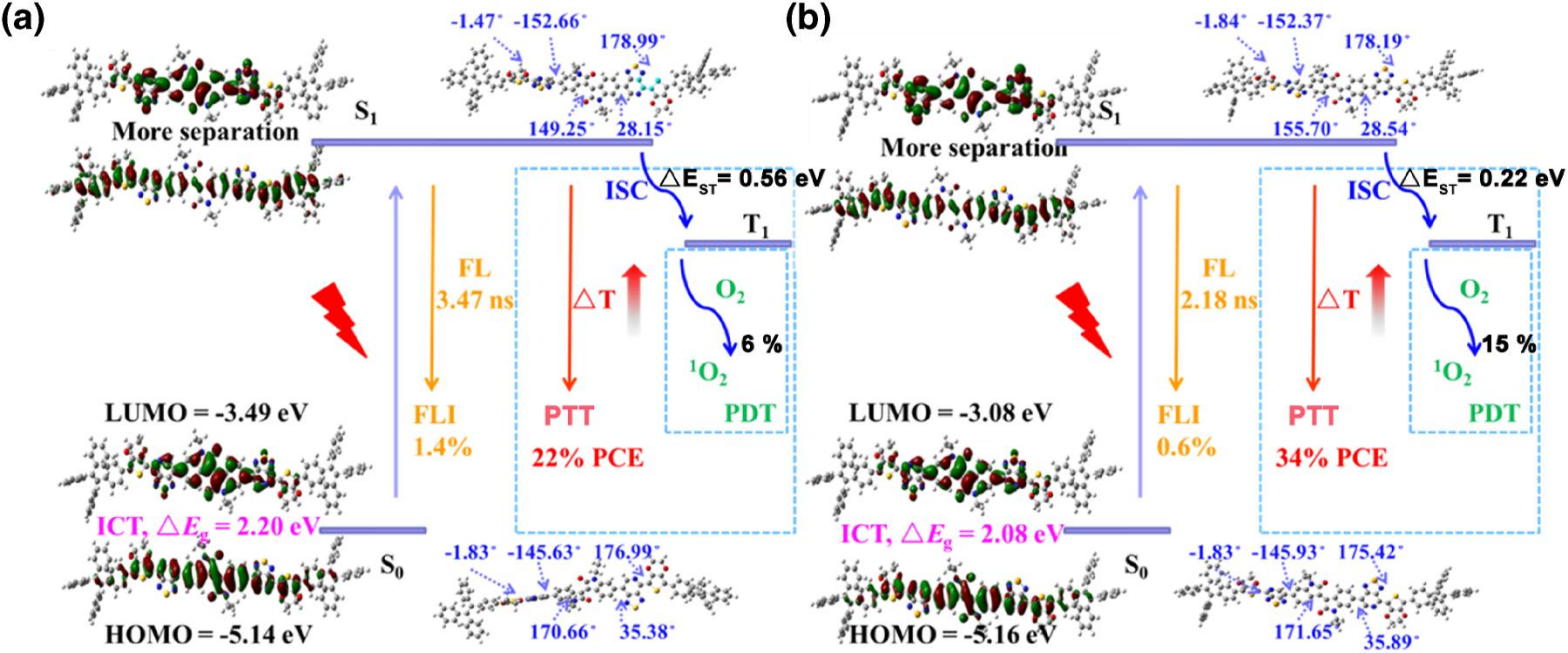
The theory model analysis of IID‐PS (a)/IID‐PSe (b) by density functional theory.

Density functional theory was used to reveal the potential mechanism and the reason for the differences between IID‐PS and IID‐PSe in photosensitization and PCE. As shown in Figure 3, IID‐PSe exhibited a lower LUMO level (−3.08 eV) than that of IID‐PS (3.49 eV), resulting in a lower HOMO‐LUMO level of 2.08 eV. What's more, the energy level (△E_ST_) of IID‐PSe (0.22 eV) was also significantly lower than that of IID‐PS (0.56 eV), indicating the energy on S_1_ state of IID‐PSe was more prone to ISC process, which was consistent with the results of ^1^O_2_ yields. The replacement of the Se atom quenched the fluorescence (from 1.4% to 0.6%), shortened the fluorescence lifetime (from 3.47 to 2.18 ns) and enhanced the PCE for IID‐PSe. It is well known that aggregation‐induced emission luminogens (AIEgens) have twisted intramolecular charge transfer (TICT) effects due to their inherent twisted structures and plentiful motion moieties, which can flexibly regulate diverse energy consumption pathways, especially for ISC process and PCE.[^1^, ^37^, ^38^] Thus, the dihedral angles of IID‐PS and IID‐PSe in the S0 state and S1 state were measured, respectively, and it was found to be basically consistent in the same state and the corresponding position, which proved that there was no TICT effect in IID‐PS and IID‐PSe. This evidence also showed that the enhancement of ^1^O_2_ yields and PCE were directly related to the spin‐orbit coupling effect of heavy atoms (Se).

**FIGURE 4:**
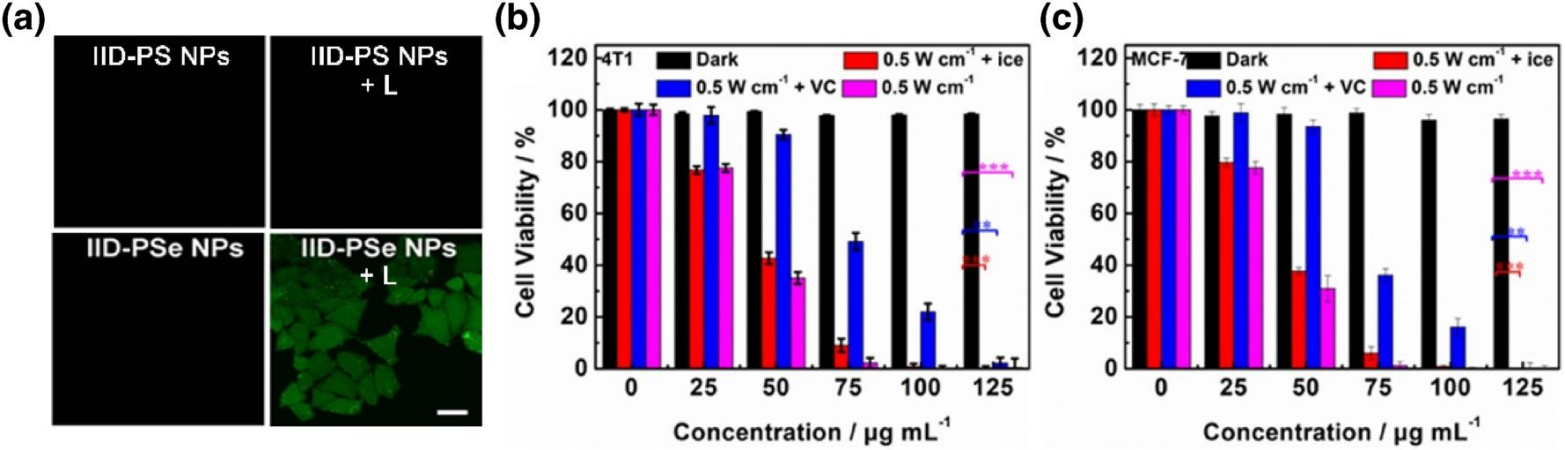
(a) Intracellular ^1^O_2_ detection in MCF‐7 cells with DCFH‐DA as an indicator under different treatments. Scale bar: 20.0 µm; NPs concentration: 25.0 μg ml^−1^; excitation: 671 nm; power: 50.0 mW cm^−2^; time: 10 min. (b) and (c) MTT analyses of the phototoxicity and dark toxicity of IID‐PSe NPs at different conditions in 4T1 and MCF‐7 cells, respectively. **P < 0.01, ***P < 0.001 determined by Student's *t*‐test.

To verify the killing cell ability in vitro, the intracellular ^1^O_2_ generation capacities of IID‐PSe NPs and IID‐PSe NPs were assessed. Two NPs were incubated with MCF‐7 cells to achieve maximum intracellular enrichment in 4 h, respectively. Then, four groups of MCF‐7 cells were treated with PBS, PBS + L (laser radiation), IID‐PS NPs/IID‐PSe NPs and IID‐PS NPs/IID‐PSe NPs + L, respectively. After adding ^1^O_2_ indicator DCFH‐DA, as shown in Figure 4a and S18, no green fluorescence was observed in the PBS and PBS + L group cells under a laser scanning confocal microscope. The cells treated by IID‐PSe NPs + L emitted bright green fluorescence, whereas IID‐PS NPs + L treated cells were almost unobservable, which indicated that IID‐PSe NPs could generate ^1^O_2_ well after entering cells instead of IID‐PS NPs. This conclusion was consistent with the results in the previous experiment (Figure S13). In Figure 4b,c, IID‐PSe NPs have no dark toxicity for cells, exhibiting outstanding biosafety. On the contrary, upon the IID‐PSe NPs concentration increasing, the cell activity gradually decreased under laser irradiation, showing excellent tumor killing ability. Of course, by removing the PTT effect with ice or removing the PDT effect with VC, the reserved single‐modal therapy of IID‐PSe NPs also showed good cell killing ability with the half fatality rate (IC50) of 44 μg mL^−1^ under single‐modal PDT and 74 μg mL^−1^ under single‐modal PTT, respectively.

In view of the excellent phototheranostic ability, IID‐PSe NPs were applied to the phototherapy of tumor‐bearing mice. At first, the fluorescence imaging performance of IID‐PSe NPs in mice was investigated. When the tumor volume reached 100 mm^3^, 150.0 μL IID‐PSe NPs solution (2.0 mg mL^−1^) was injected intravenously. The enrichment of IID‐PSe NPs in mice tumor tissue was observed by fluorescence imaging under 820 nm filter. With the extension of time, the fluorescence intensity in the tumor area gradually increased and reached the maximum value at 9 h, while the fluorescence signal in other areas could be ignored, as shown in Figure S19. After that, the fluorescence gradually weakened with the passage of time, and basically disappeared after the 18th h, indicating that the nanoparticles were metabolized at this time. After the mice were euthanized and their major organs were dissected, the fluorescence was found to be in tumor sites followed by the liver and kidney, indicating that the nanoparticles are mainly metabolized through the liver and kidney.

**FIGURE 5:**
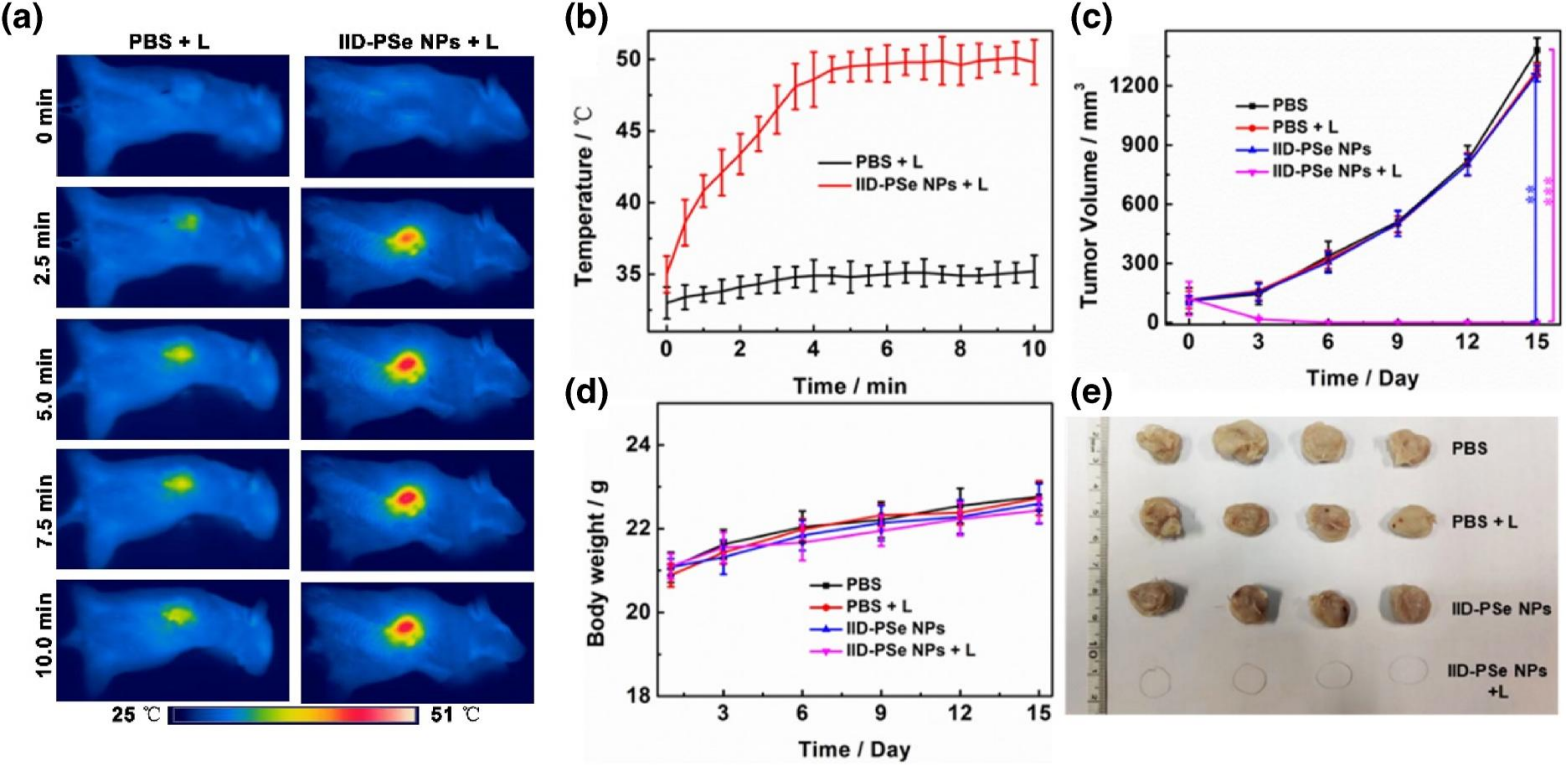
(a) Photothermal images of 4T1‐tumor‐bearing nude mice after various treatments. (b) Temperature changes in the tumor region under laser irradiation. (c) The changes of tumor volume in different groups. (d) The changes of mice weight in different groups. (e) Photographs of tumor after 15 days. **P < 0.01, ***P < 0.001 determined by Student's *t*‐test.

Then the tumor sites of mice were subjected to laser irradiation (671 nm, 0.5 W cm^−2^) at 9th h after injection of IID‐PSe NPs (150.0 μL, 2.0 mg mL^−1^) into the tail vein. During laser irradiation, the temperature of tumor site in the phototherapy group rose rapidly to 48°C within 10 min, while that in the blank group and control group remained basically unchanged (Figure 5a,b, and Figure S20). After laser irradiation, all mice were monitored for changes in body weight and tumor volume every day for 15 days. On the third day, the tumor of mice in the phototherapy group basically disappeared, and there was no recurrence within 15 days (Figures 5c,e). In contrast, tumor volume increased 12‐fold on day 15 in mice in the blank and control groups, respectively. In addition, the weight gain of the four groups of mice was basically consistent (Figure 5d), indicating that IID‐PSe NPs have basically no side effects on mice and have good biological safety.

**FIGURE 6:**
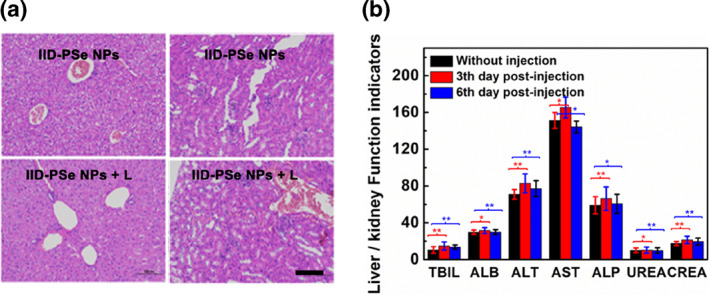
Hematoxylin and eosin (H&E) of liver (a, left) and kidney (a, right) treated with different conditions. Scale bar = 200 μm. (b) Hematological assessment of liver and kidney function indicators of mice with different treatments. Unit: TBIL: μg mL^−1^; ALB: g L^−1^; alanine aminotransferase (ALT)/aspartate aminotransferase (AST)/ALP: U L^−1^; UREA: mmol L^−1^; CREA: μmol L^−1^. *P < 0.05, **P < 0.01 determined by Student's *t*‐test.

In Figure 6a and S21, tissue sections of the phototherapy group of mice were similar to those in the blank group and the control group, and no tissue damage occurred. However, there was a significant difference for tumor tissue. IID‐PSe NPs + L led significant damage to the tumor tissue, and tumor tissue cells appeared necrosis and other characteristics. Next, since IID‐PSe NPs are metabolized primarily by the liver and kidneys, we studied the changes of liver function markers total bilirubin, plasma albumin, alanine aminotransferase, aspartate aminotransferase and alkaline phosphatase, as well as the renal function markers UREA (urea) and CREA (creatinine) before treatment, 3th days after treatment, and 6th days after treatment, respectively.^[39]^ As shown in Figure 6b, all liver function markers showed no abnormal changes before and after treatment and were within the normal range. Finally, the whole blood of the four groups of mice was extracted for blood composition analysis (Table S1). Blood cells (including white blood cells, lymphocytes, neutrophils, erythrocyte hemoglobin, and platelet count) showed no abnormal changes before and after treatment, maintaining within the normal range. The above data indicated that IID‐PSe NPs did not cause side effects in mice during treatment and had excellent biological safety.

## 3 CONCLUSION

In summary, we presented a dual‐acceptor engineering strategy to across‐the‐board improve anti‐tumor phototherapy of D‐A type photosensitive dye. A series of D‐A type photosensitive dyes (PS, IID‐PS, IID‐PSe) based on this strategy were constructed with IID, sulfur or selenium‐substituted [1,2,5]thiadiazolo[3,4‐*c*]pyridine as acceptors and 3,4‐ethylenedioxythiophene‐tetraphenylethylene as donor. Among them, IID‐PSe has the highest molar extinction coefficient, strengthened ICT effect and spin‐orbit coupling effects. So that, IID‐PSe exhibited weak fluorescence properties, strong photon‐absorption ability (2 times than that of PS), and decreased HOMO‐LUMO energy level and △E_ST_, achieving synchronous improvement of PDT and PTT with ^1^O_2_ yield of 15% and PCE of 34%. After DSPE‐PEG2000 encapsulation, IID‐PSe NPs showed excellent PDT and PTT combined phototherapy ablation tumor ability both in vitro and in vivo, whereas they have good biological safety for major tissues and organs of mice. We expect this work to provide a new avenue for designing simultaneously enhanced phototherapy agents with high photon‐absorption ability, ROS yield and PCE.

## CONFLICT OF INTEREST STATEMENT

The authors declare no conflicts of interest.

## ETHICS STATEMENT

All animal experiments were performed according to the guide lines of the Care and Use of Laboratory Animals formulated by the Ministry of Science and Technology of China and were approved by the Animal Care and Use Committee of Dalian University of Technology (2019–018).

## Supporting information

Supplementary Material

## Data Availability

Supporting Information is available from the Wiley Online Library or from the author.
