# Supplementary material for: Dual‐acceptor engineering of donor‐acceptor type molecules for all‐round boosting anti‐tumor phototherapy
Source: Smart Mol. 2023 Nov 23;2(2):e20230014. doi: 10.1002/smo.20230014 (PMC12118229; doi:10.1002/smo.20230014)
Supplement: Supplementary file 1 — Supplementary Material [file SMO2-2-e20230014-s001.docx]

Supporting Information

Dual-acceptor engineering of D-A type molecules for all-round boosting anti-tumor phototherapy

Hua Gu^1,2^, Wen Sun^1,2^, Jianjun Du^1,2^, Jiangli Fan^1,2,*^, Xiaojun Peng^1^

^1^ State Key Laboratory of Fine Chemicals, Frontier Science Center for Smart Materials, Dalian University of Technology, Dalian 116024, China.

^2^ Ningbo Institute of Dalian University of Technology, Ningbo 315016, China.

*Materials*

The chemical intermediates of diphenyl-methan, 4-bromobenzophenone, tributyl(2,3-dihydrothieno[3,4-b][1,4]dioxin-5-yl)stannane, 4,7-dibromo-[1,2,5]thiadiazolo[3,4-c]pyridine, 4,7-dibromo-[1,2,5]selenadiazolo[3,4-c]pyridine, (e)-6,6'-dibromo-n,n'-bis(5-decylpentadecyl)-isoindigo, palladium catalyst, 3,(4,5-dimethylthiazol-2-yl)-2,5-diphenyl tetrazolium bromide (MTT), 1,3-diphenylisobenzofuran (DPBF), and 9,10-anthracenyl-bis(methylene)-dimalonic acid (ABDA) were commercially available. The solvents were ordered from Xilong Scientific and Damao Chemical Reagent. Dulbecco’s modified eagle medium (DMEM), phosphate-buffered saline (PBS), fetal bovine serum (FBS), penicillin, and streptomycin (P/S) were purchased from Beijing Solarbio Science & Technology Co., Ltd; Reactive oxygen species assay kit (DCFH-DA) and calcein AM/propidium (AM/PI) were purchased from Keygen Biotechnology.

This study was conducted in accordance with the Guide for the Care and Use of Laboratory Animals published by the US National Institutes of Health (8th edition, 2011). The animal protocol was approved by the local research ethics review board of the Animal Ethics Committee of Dalian University of Technology.

Synthesis

The synthesis process of each intermediate product and final compound was described as follows:


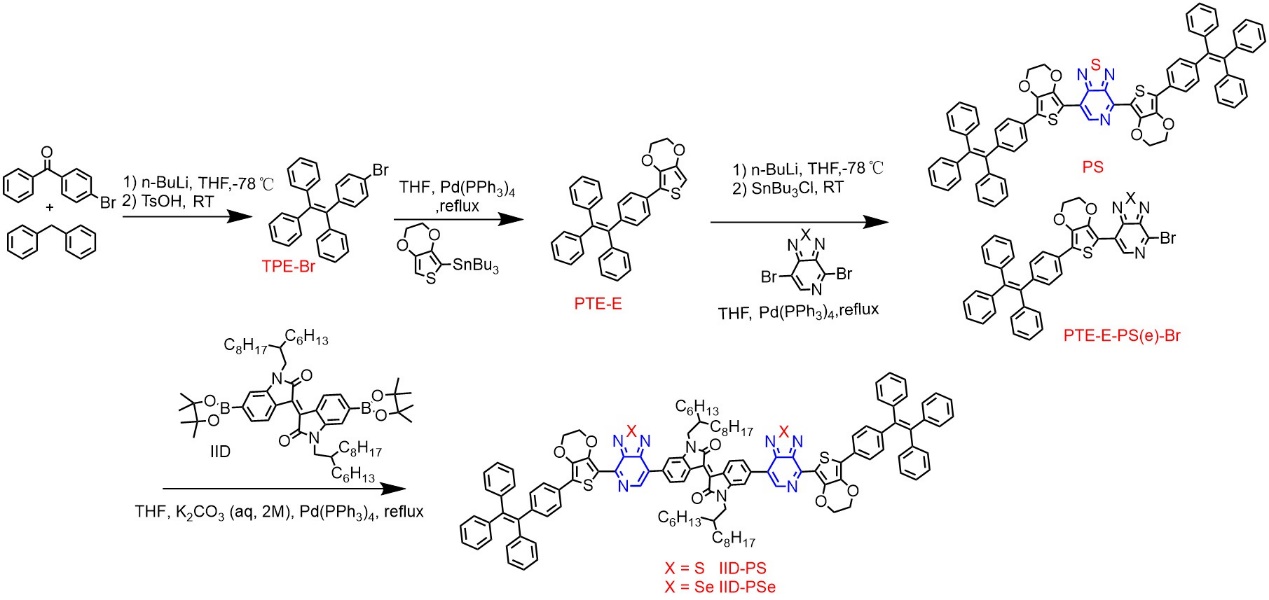


Figure S1 Synthetic routes of PS, IID-PS and IID-PSe

TPE-Br：In the absence of oxygen, diphenyl methane (2.0 g, 12.0 mmol) was dissolved in ultra-dry tetrahydrofuran (20.0 mL); The reaction temperature was maintained at -78℃, and n-butyl lithium (4.0 mL, 10.0 mmol, 2.5 M in Hexane) was added drip by drop. Stir continuously for 30 min to obtain an orange-red solution. Then 4-bromobenzophenone (3.1 g, 12.0 mmol) was added drip by drop into the mixture. After drip, the temperature was restored to room temperature, and the mixture was stirred continuously for 6 h. The reaction was quenched by adding saturated ammonium chloride aqueous solution (10.0 mL), and the organic layer was extracted by methylene chloride (10.0 mL). The organic layer was rinsed with saturated brine, and the anhydrous magnesium sulfate was dried. Evaporate the solvent, obtain the crude product, and dissolve it into 80 mL toluene. A catalytic amount of p-toluene sulfonic acid (0.3 mg, 1.8 mmol) was added into the reaction system, and the mixture was refluxed for 34 h and cooled to room temperature. The toluene layer was washed with 10% sodium bicarbonate aqueous solution (30.0 mL) and dried with anhydrous magnesium sulfate for 12 h. The anhydrous magnesium sulfate was filtered and the filtrate was rotated dry to obtain the crude tetraphenylvinyl derivatives. Using n-hexane as eluent, the crude product was purified by silica gel column to obtain white solid. The yield was as follows: 93.6%。^1^H NMR (400 MHz, DMSO-*d*_6_) *δ* 7.46 (d, *J* = 15.4 Hz, 8H), 7.32 (d, *J* = 7.5 Hz, 2H), 7.17 – 6.97 (m, 9H), 6.07 (s, 1H), 5.23 (s, 1H)；^13^C NMR (101 MHz, DMSO-*d*_6_) *δ* 147.25, 147.01, 142.33, 130.42, 130.05, 128.74, 127.54, 127.22, 126.33, 125.96, 125.47, 119.03。

PTE-E：In the absence of oxygen, tetraphenyl bromide (0.3 g, 1.0 mmol) and 2-tributylstany-3, 4-ethylenedioxythiophene (0.4 g, 1.1 mmol) were dissolved in ultra-dry tetrahydrofuran (10.0 mL). Tetraphosphine palladium (Pd(PPh3)4, 0.2 g, 0.2 mmol) catalyst was added to the mixture and the reaction was carried out at 85℃ for 12 h. The reaction progress was monitored with a point plate. When the bromotetraphenylvinyl reaction was complete, the reaction was stopped and cooled to room temperature. The mixture was washed with saturated salt water (20.0 mL), extracted with methylene chloride (5.0 mL), and dried with anhydrous magnesium sulfate for 12 h. Finally, the anhydrous magnesium sulfate was filtered out, the filtrate was dried, and the cake was separated and purified by silica gel column (n-hexane: dichloromethane = 4:1) to obtain the target product. PTE-E: yellow green solid, yield 68.3%。^1^H NMR (400 MHz, CDCl_3_-*d*_6_) *δ* 7.47 (d, *J* = 7.9 Hz, 2H), 7.05 (dt, *J* = 16.0, 9.0 Hz, 17H), 6.25 (s, 1H), 4.27 (d, *J* = 4.3 Hz, 2H), 4.23 – 4.20 (m, 2H)；^13^C NMR (100 MHz, CDCl_3_-*d*_6_) *δ* 144.02, 143.61, 142.23, 141.97, 140.94, 140.61, 138.19, 131.70, 131.25, 127.89, 127.51, 126.58, 126.27, 124.95, 117.48, 97.48, 64.58, 64.41, 64.32。

PS: PTE-E (0.4g, 1.0 mmol) was dissolved in 20.0 mL ultra-dry tetrahydrofuran. Cool the unit to -80°C and protect it with nitrogen. N-butyllithium (n-BuLi, 7.0 mL, 1.2 mmol, 1.6 M) was added drop by drop into the reaction system, and kept stirring at -80°C for 3 h. Subsequently, tributyl tin chloride (SnBu3Cl, 0.4g, 1.2mmol) was added to the reaction system drop by drop. After the reaction system temperature was slowly restored to room temperature, the stirring reaction was continued for 12 h. When the reaction was over, the mixture was poured into saturated salt water (20 mL) and extracted with ethyl ether (10 mL). The operation was repeated three times. The extract was dried with anhydrous magnesium sulfate and left for 6 h. Anhydrous magnesium sulfate is filtered, the filtrate is dried without any other treatment, and directly used for the next reaction.

Tin butyl PET-E (6.0 g, 2.5 mmol), 4, 7-dibromo-1,2, 5-benzothiadiazole (0.3 g, 1.1 mmol), [bis (diphenylphosphine) ferrocene] Palladium dichloride (Pd(dppf)2Cl2, 0.03 g, 0.05mmol) was added into the double-neck flask. Reaction system nitrogen protection. Toluene (20 mL), a refined solvent, was added and reacted at 100℃ for 12 h. After the reaction was over, the mixture was poured into saturated salt water (50.0 mL), and methylene chloride (10.0 mL) was extracted, repeated three times. Appropriate amount of anhydrous magnesium sulfate was added into the extraction solution and dried for 6 h. The anhydrous magnesium sulfate was filtered, the filtrate was dried as solvent, and the cake was separated and purified by silica gel column (n-hexane: dichloromethane = 2:1).

PS: Purple-black solid, yield 82.1%. ^1^H NMR (400 MHz, CDCl_3_-*d*_6_) *δ* 9.35 (s, 1H), 7.63 (dd, *J* = 20.1, 6.1 Hz, 4H), 7.07 (dd, *J* = 33.1, 16.5 Hz, 34H), 4.48 (d, *J* = 53.7 Hz, 8H); ^13^C NMR (100 MHz, CDCl_3_-*d*_6_) *δ* 158.79, 143.69, 141.47, 140.93, 138.17, 131.55, 127.96, 127.43, 126.74, 125.96, 125.68, 125.33, 119.71, 65.34, 64.73, 64.40.

The synthesis methods of PET-PS-Br and PET-PSe-Br refer to the synthesis steps of PS, and the different variables are that the molar ratio of stutylated PET-E to 4, 7-dibromo-1,2, 5-benzothiol/selenodiazole is 1.2:1.

PET-PS-Br: yields of 52.6%. ^1^H NMR (400 MHz, CDCl_3_-*d*_6_) *δ* 8.74 (d, *J* = 8.0 Hz, 1H), 7.64 (d, *J* = 8.3 Hz, 2H), 7.08 (ddd, *J* = 18.5 Hz, 17H), 4.49 (d, *J* = 4.3 Hz, 2H), 4.41 (d, *J* = 4.2 Hz, 2H); ^13^C NMR (100 MHz, CDCl_3_-*d*_6_) *δ* 146.19, 144.57, 143.60, 141.52, 138.66, 131.77, 131.54, 131.23, 130.33, 127.96, 127.54, 126.84, 126.32, 125.78, 65.29, 64.30.

PET-PSe-Br: yields of 47.4%. ^1^H NMR (400 MHz, CDCl_3_-*d*_6_) *δ* 8.62 (s, 1H), 7.67 (d, *J* = 8.2 Hz, 2H), 7.18-7.01 (m, 17H), 4.48 (d, *J* = 33.7 Hz, 4H); ^13^C NMR (100 MHz, CDCl_3_-*d*_6_) *δ* 146.17, 143.62, 131.72, 131.39, 130.04, 127.45, 126.77, 126.23, 125.79, 65.31, 64.29.

IID-PS / IID-PSe: Toluene (20.0 mL), PET-PS-Br/ PET-PSe-Br (1.5/1.6 g, 2.2 mmol), 6,6' -borate -N,N'-(2-hexyloctane) -iso-indigo-blue (1.0 g, 1.1 mmol) and 2 M potassium carbonate aqueous solution (2.5 mL) were mixed in two-necked flask. And then, Pd(dppf)_2_Cl_2_ (0.06 g, 10% mmol) was added under nitrogen protection. The mixture was refluxed for 20 h. After the complete reaction, the reaction was stopped and restored to room temperature. The mixture was washed in saturated salt water (50 mL) and extracted three times with CH_2_Cl_2_ (3×10.0 mL). Appropriate amount of anhydrous magnesium sulfate was added into the extraction solution and dried for 6 h. The anhydrous magnesium sulfate was filtered out, the filtrate was removed the solvent, and the residual solid was separated and purified by silica gel column (n-hexane: dichloromethane = 1:1).

IID-PS: Blue-purple solid, yields of 38.2%. ^1^H NMR (400 MHz, CDCl_3_-*d*_6_) *δ* 9.35 (s, 1H), 8.74 (d, *J* = 8.0 Hz, 1H), 7.66 (d, *J* = 8.3 Hz, 8H), 7.49 (d, *J* = 24.1 Hz, 4H), 7.09 (d, *J* = 16.9 Hz, 30H), 4.58 (s, 4H), 4.43 (s, 4H), 3.80 (s, 4H), 2.40 (s, 2H), 1.26 (s, 48H), 0.83 (s, 18H); ^13^C NMR (100 MHz, CDCl_3_-*d*_6_) *δ* 167.22, 165.27, 156.02, 143.58, 132.27, 132.17, 131.98, 131.39, 128.85, 128.55, 128.32, 127.29, 126.57, 125.83, 122.04, 108.83, 65.50, 64.38, 36.46, 31.84, 30.15, 29.58, 29.20, 28.92, 26.78, 26.51, 22.63, 14.0.

IID-PSe: black solid, yields of 35.8%. ^1^H NMR (400 MHz, CDCl_3_-*d*_6_) *δ* 9.33 (s, 1H), 8.89 (s, 1H), 7.58 (dd, *J* = 60.6, 18.8 Hz, 12H), 7.08 (d, *J* = 19.4 Hz, 30H), 4.62-4.27 (m, 8H), 3.79 (s, 4H), 2.03 (s, 2H), 1.45-1.21 (m, 48H), 0.83 (s, 18H); ^13^C NMR (100 MHz, CDCl_3_-*d*_6_) *δ* 168.52, 155.70, 148.79, 147.63, 145.64, 144.56, 143.89, 142.49, 141.45, 140.44, 138.72, 132.11, 131.77, 131.41, 130.45, 128.50, 128.07, 127.46, 126.61, 125.77, 125.01, 121.89, 112.31, 108.76, 65.46, 64.31, 44.81, 36.46, 31.82, 30.59, 30.15, 29.69, 29.31, 26.65, 22.65, 19.19, 14.09.

Nanoparticles preparation

The preparation process was as follows: 1.0 mg of IID-PS or IID-PSe and 5.0 mg of DSPE-PEG200 were added to 1.0 mL of tetrahydrofuran (THF), and the mixture was sonicated in an ultrasonic cleaning instrument for 15 min. 9.0 mL deionized water was sonicated in an ultrasonic cell shatter (φ2.5 cm, 25% W), and the THF solution was quickly added to the deionized water at one time and continuously sonicated for 10 min. The prepared crude nanoparticles were dialyzed in a dialysis bag (3500) for 48 hours, and the water was changed every 6 hours during the dialysis. IID-PS NPs or IID-PSe NPs with uniform particle size were prepared by filtering the crude product through a 220 nm filter membrane. Finally, the nanoparticles were concentrated by centrifugation in a centrifuge (3000 rpm, 10 min), and the concentrated IID-PS/IID-PSe NPs solution was directly used as mother liquor for subsequent experiments after calibration.

**Characterization of NPs**. The size and morphology of NPs were acquired with DLS (Malvem Zetasizer Nano ZS) and TEM (Philips Technai 12), respectively. The absorption data were obtained by UV−vis spectrophotometer (Shimadzu 1700). The fluorescence quantum yield was determined from a UV−NIR Absolute PL quantum yield spectrometer.

**Photothermal conversion efficiency calculation**

In order to explore the relationship between nanoparticle concentration and temperature change, the quantified nanoparticles (1.0 mg mL^-1^) were diluted to different concentrations from 50.0 μg mL^-1^ to 200.0 μg mL^-1^. The 671 nm laser with 0.5 W cm^-2^ optical power density was used to radiate the above concentration of nanoparticles solution for 10.0 min, and the temperature at different time points was recorded by a thermal imaging camera. To test whether the temperature change depends on the optical power density, The optical power densities from 0.5 W cm^-2^ to 0.1 W cm^-2^ under the wavelength of 671 nm laser were selected to radiation 1.5 mL of IID-PS NPs/IID-PSe NPs (100.0 μg mL^-1^), respectively. The temperature at different time points was also recorded with a thermal imaging camera.

The photothermal conversion efficiencies (η) were measured according to the reported method:

$$\boldsymbol{\eta}\mathbf{=}\frac{\mathbf{hs}\left( \mathbf{T}_{\mathbf{Max}}\mathbf{-}\mathbf{T}_{\mathbf{Surr}} \right)\mathbf{-}\mathbf{Q}_{\mathbf{Dis}}}{\boldsymbol{I}\mathbf{(1-}\boldsymbol{10}^{\boldsymbol{-A}\boldsymbol{671}}\mathbf{)}}$$

h is the heat transfer coefficient; s is the surface area of the container. Q_Dis_ represents heat dissipated from the laser mediated by the solvent and container. I is the laser power and A is the absorbance at 671 nm.

$$\mathbf{hs=}\frac{\boldsymbol{mC}_{\boldsymbol{water}}}{\boldsymbol{\tau}}$$

m is the mass of the solution containing the photoactive material, C is the specific heat capacity of the solution (C_water_ = 4.2 J/(g•°C)), and τ is the associated time constant.

$$\boldsymbol{t=-\tau ln\theta}$$

θ is a dimensionless parameter, known as the driving force temperature

$$\boldsymbol{\theta=}\frac{\mathbf{T-}\mathbf{T}_{\mathbf{Surr}}}{\mathbf{T}_{\mathbf{Max}}\mathbf{-}\mathbf{T}_{\mathbf{Surr}}}$$

T_max_ and T_Surr_ are the maximum steady state temperature and the environmental temperature, respectively.

UV−vis spectrophotometer test

1.0 mg IID-PS/IID-PSe CH_2_Cl_2_ solution into a 5.0 mL, 0.2 mg mL^-1^ mixture solution. 0.3 mL of the solution was removed and added to a quartz dish containing 3.0 mL, and the absorbance was measured under ultraviolet absorption spectrum. Similarly, 0.2 mL mother solution of IID-PS/IID-PSe NPs (1.0 mg mL^-1^) was removed and diluted to 20.0 μg mL^-1^ with 5.0 mL deionized water, and their absorbance was measured under ultraviolet absorption spectrum.

^1^O_2_ detection

The ability of IID-PS/IID-PSe to produce singlet oxygen was tested in a dichloromethane system. The concentration standard was formulated as follows: the absorbance of DPBF dichloromethane solution at 420 nm was adjusted to between 1.0 and 1.2, while the absorbance of the maximum absorption peak of IID-PS/IID-PSe was controlled to about 0.3 to 0.4. The laser was used at 671 nm, the optical power density was 20.0 mW cm^-2^, and the illumination time was 10 min. The absorption spectra were tested at 2 min intervals.

ABDA was selected as the probe to detect ^1^O_2_ generation in PBS. Briefly, the mixtures of ABDA and NPs in PBS were irradiated under a 671-nm laser (50.0 mW cm^-2^). The degradation of absorbance of ABDA was recorded by a UV–vis–NIR spectrophotometer. In addition, DCFH-DA was chosen as an intracellular ROS indicator. First, MCF-7 cells and NPs (50.0 μL, 1.0 mg mL^-1^) were coincubated for 24 h in the dark. Then, DCFH-DA (5.0 μL) was added. After 20 min coincubation, the experimental groups were irradiated under a 671-nm laser (50.0 mW cm^-2^) for 10 min. After another 30 min of coincubation, the fluorescence signal was monitored by CLSM. Finally, the control group images were obtained under the same conditions except for variables.

In vitro cytotoxicity

We verified the phototoxicity and dark toxicity of NPs for 4T1 and MCF-7 cells, respectively. Briefly, Calcein-AM/PI (5.0 μL) was added to four groups of 4T1 or MCF-7 cells treated by PBS, PBS + laser (671 nm，0.5 W cm^-2^，10 min), NPs (100.0 μL, 1.0 mg mL^-1^), and NPs (100.0 μL, 1.0 mg mL^-1^) + laser (671 nm，0.5 W cm^-2^，10 min), respectively. The final staining results were recorded by CLSM. Next, we replaced Calcein-AM/PI with MTT (0.5 mg mL^-1^) following the above procedure. Finally, the resulting formazan compound was dissolved by DMSO, and its optical absorption was detected in an enzyme-labeled instrument. Furthermore, because IID-PSe NPs has both PDT and PTT abilities, two additional control groups were added, Vitamin C (VC) to consume ROS and ice environment to prevent light-to-heat conversion.

Tumor-bearing mouse model

All in vivo studies were approved by the Dalian Medical University Animal Care and Use Committee. A total of 150.0 μL of 4T1 cells (1 × 10^7^) in PBS was inoculated in the underarm epidermis of mice (BALB/c, female, 4 weeks of age, Jilin Changsheng Biotechnology). When the tumor reached ~100 mm^3^ in size, these mice were used directly for bioimaging and phototherapy.

Fluorescence bioimaging

The excitation wavelength of the imaging system was set as 620 nm and the emission wavelength was set as 820 nm. Tumor-bearing female BALB/c mice were intraperitoneally injected with 0.1 mL chloral hydrate solution (4.0 mg mL^-1^). We injected 150.0 μL of IID-PSe NPs (1.5 mg mL^-1^) through a mouse tail vein until the tumor reached 100 mm^3^ in size, and the enrichment of IID-PSe NPs in the tumor site was observed under the in vivo imaging system.

In vivo antitumor efficiency

One phototherapy group and three control groups were established containing five tumor-bearing mice. Each mouse was injected with IID-PSe NPs (150.0 μL, 1.5 mg mL^-1^) in a tail vein. The tumor-bearing mice in the phototherapy group were exposed to the 671-nm laser (0.5 W cm^-2^) for 10 min. The other three control groups were treated with PBS only, PBS + laser (671 nm，0.5 W cm^-2^，10 min), and IID-PSe NPs only, respectively. The tumor size and bodyweight of all the mice were monitored every 3 days until 15 days had passed. Eventually, all the mice were euthanized, and their primary organs and tumors were collected to assess the biosafety of IID-PSe NPs.


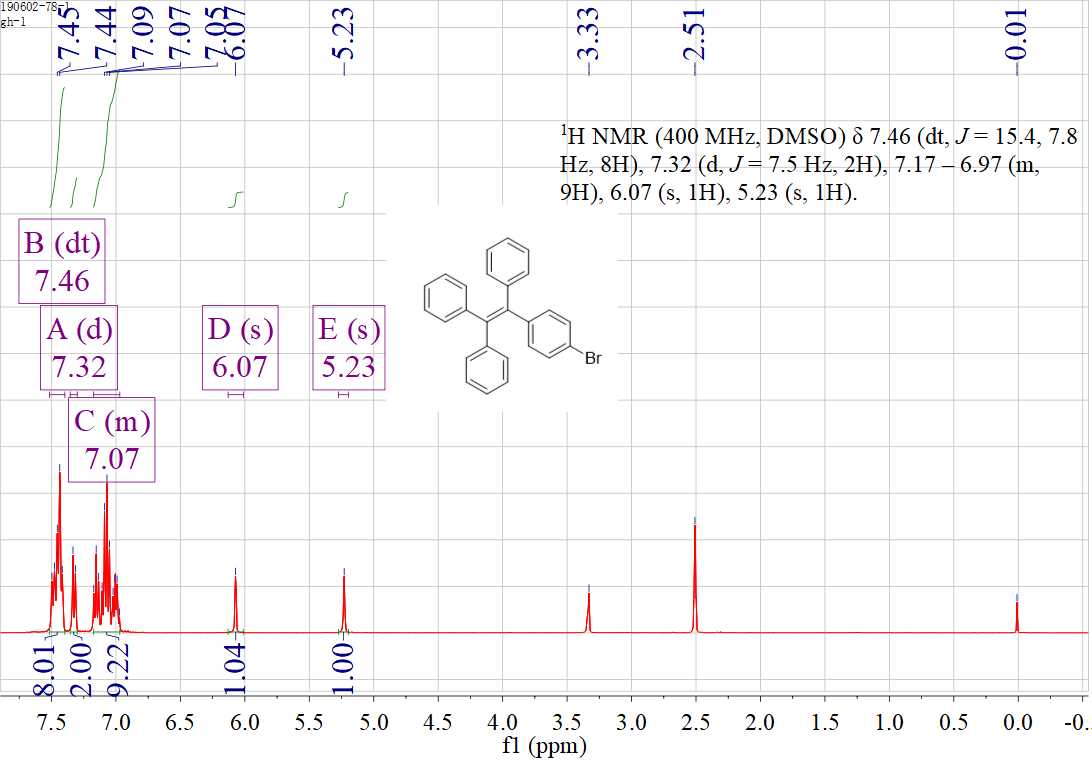


Figure S1 ^1^H NMR of PET-Br in DMSO-*d*_6_


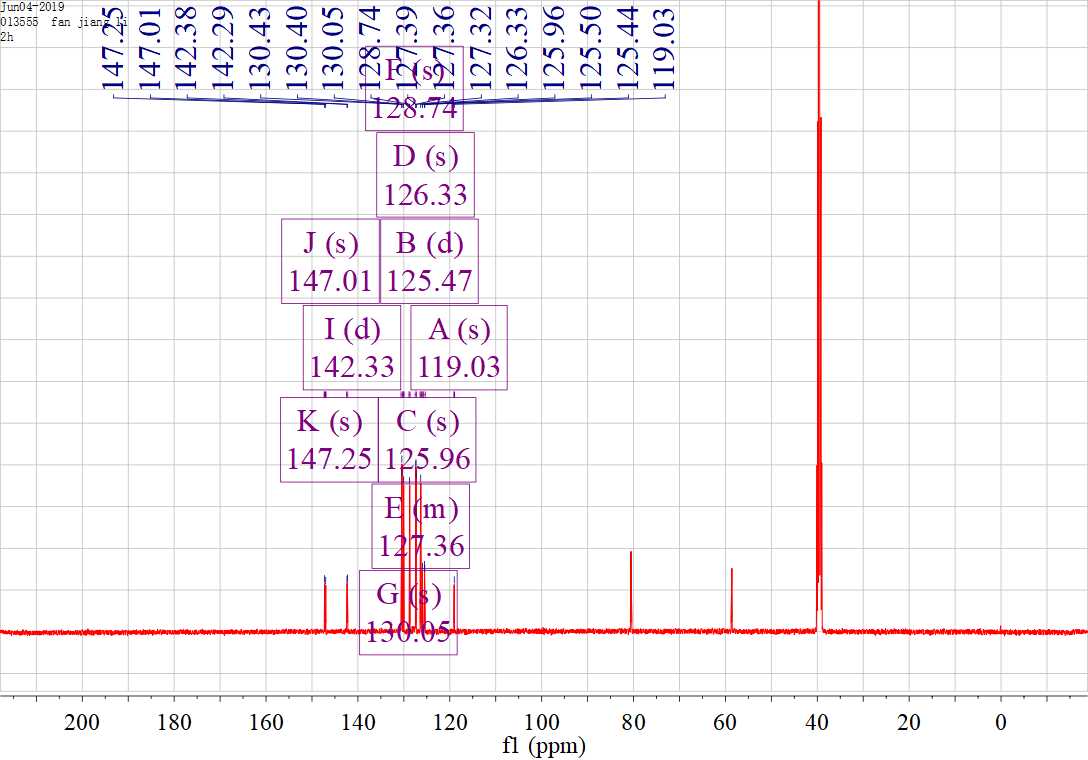


Figure S2 ^13^C NMR of PET-Br in DMSO-*d*_6_


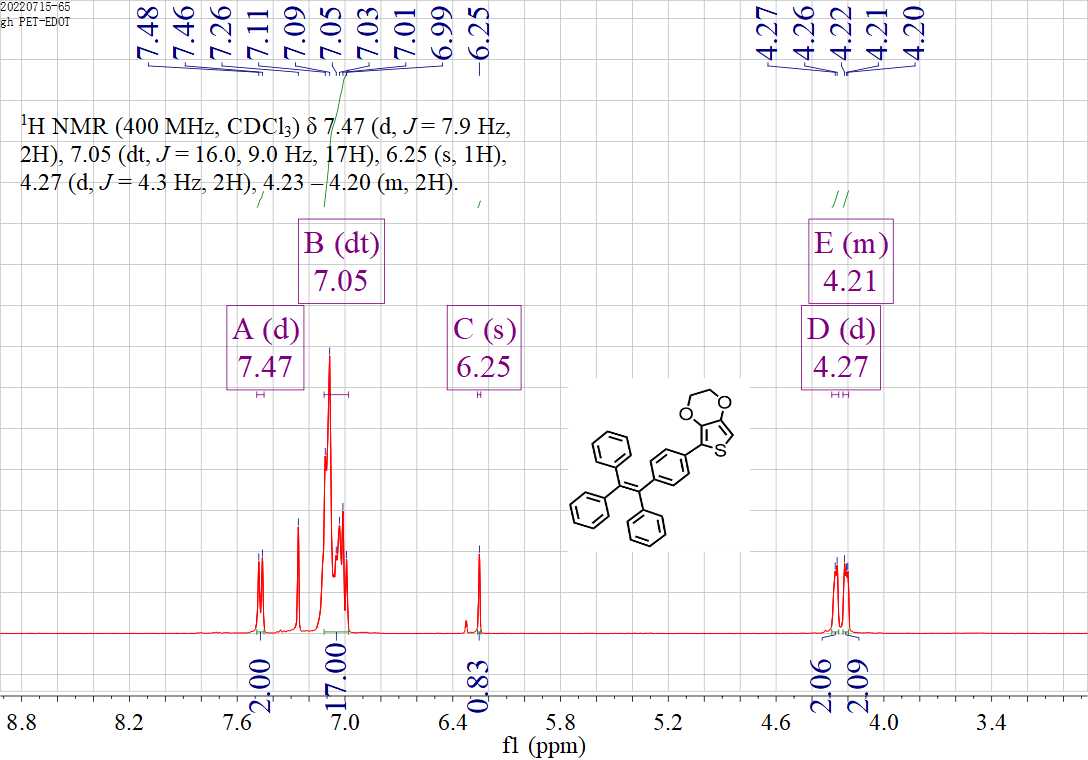


Figure S3 ^1^H NMR of PET-E in CDCl_3_-*d*_6_


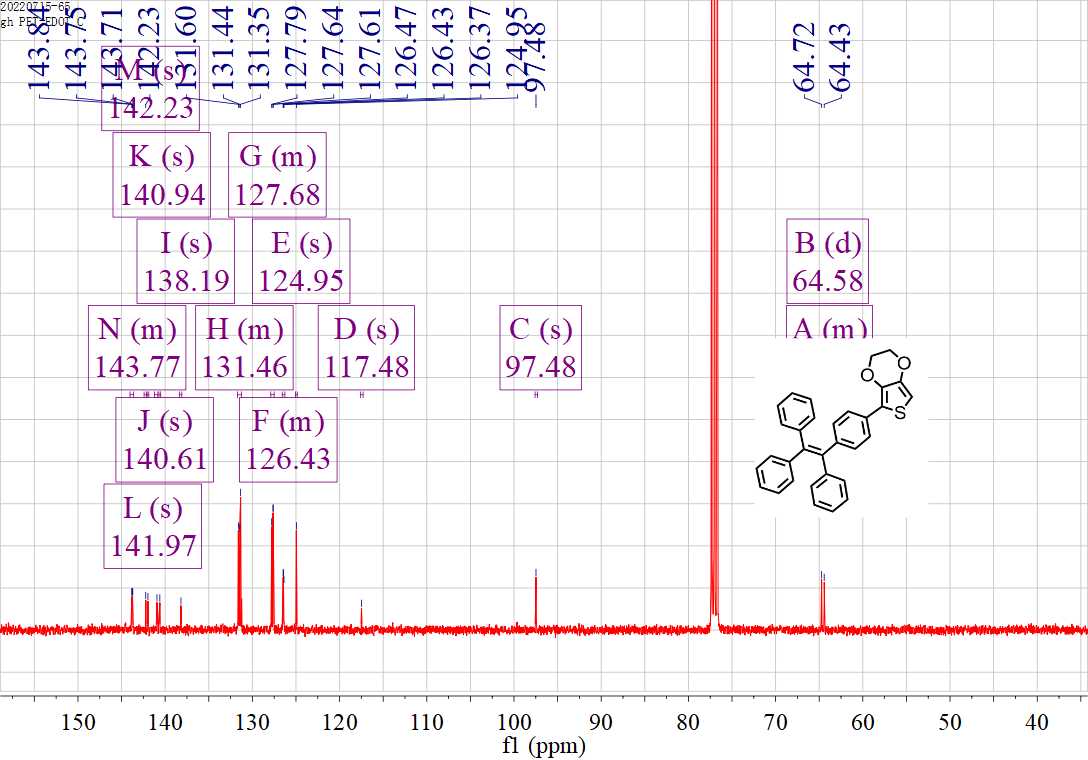


Figure S4 ^13^C NMR of PET-E in CDCl_3_-*d*_6_


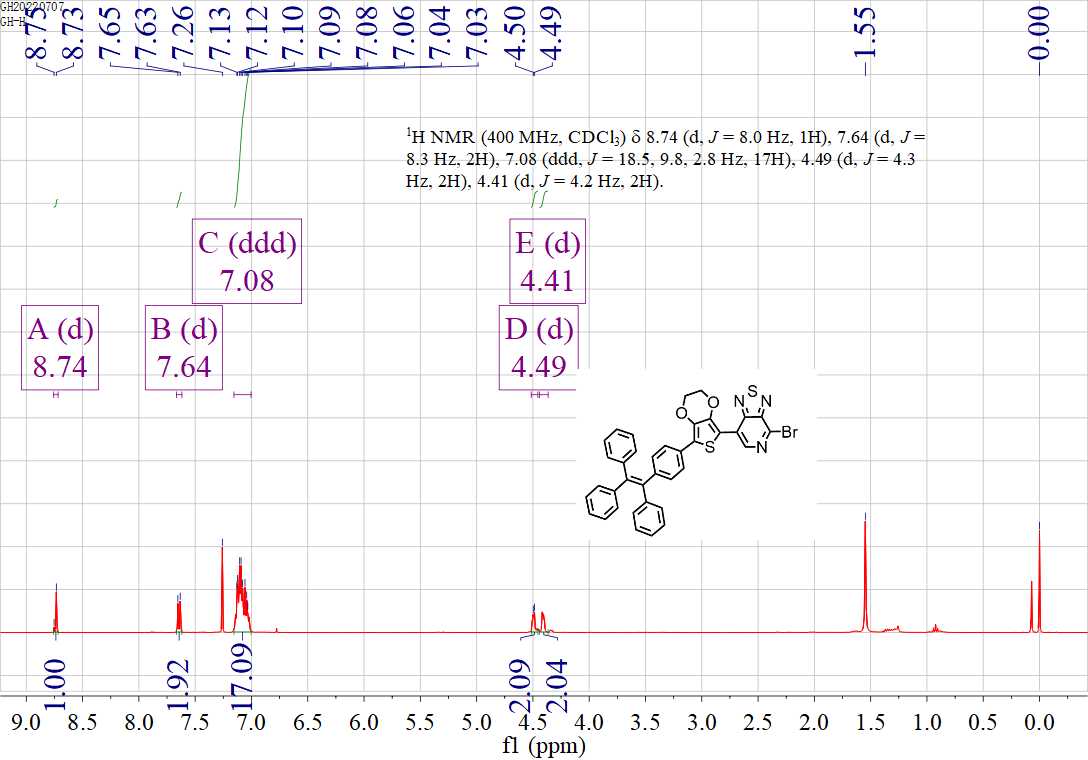


Figure S5 ^1^H NMR of PET-E-PS-Br in CDCl_3_-*d*_6_


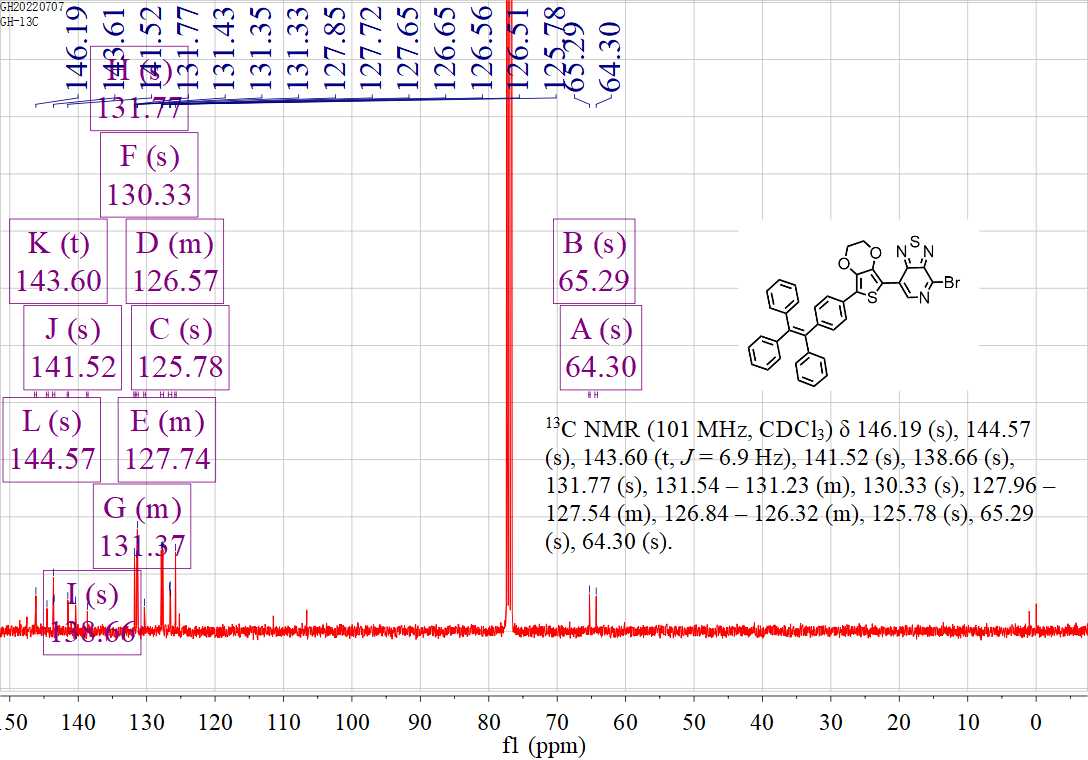


Figure S6 ^13^C NMR of PET-E-PS-Br in CDCl_3_-*d*_6_


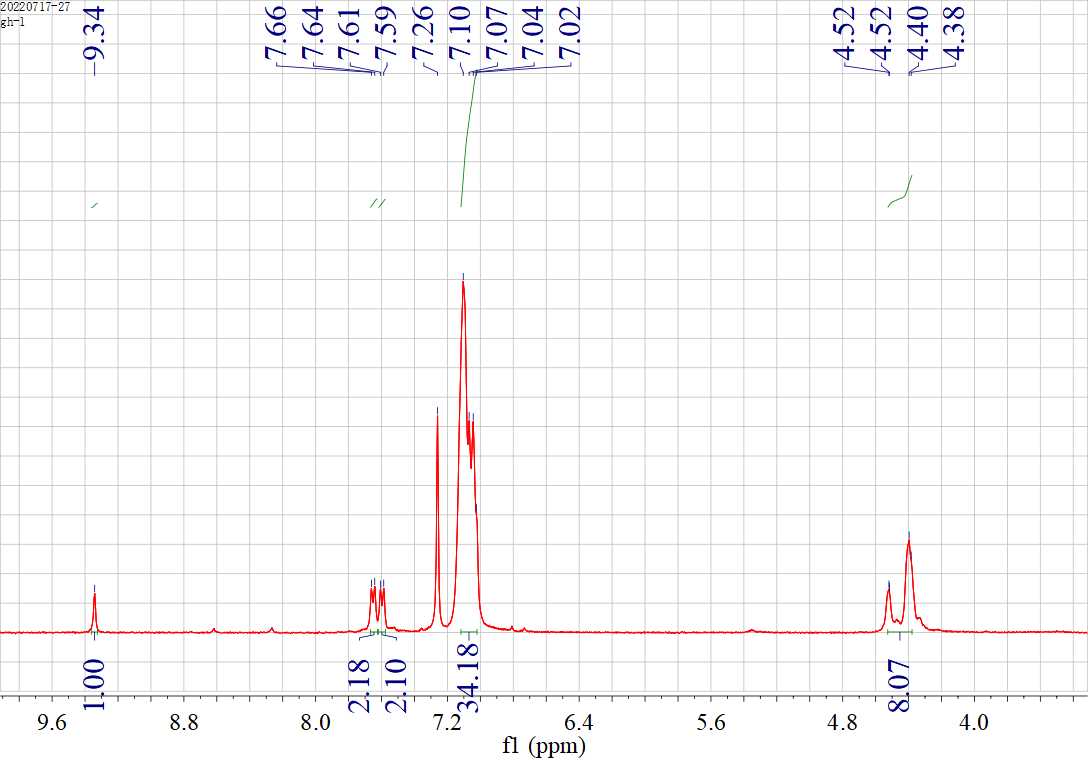


Figure S7 ^1^H NMR of PS in CDCl_3_-*d*_6_
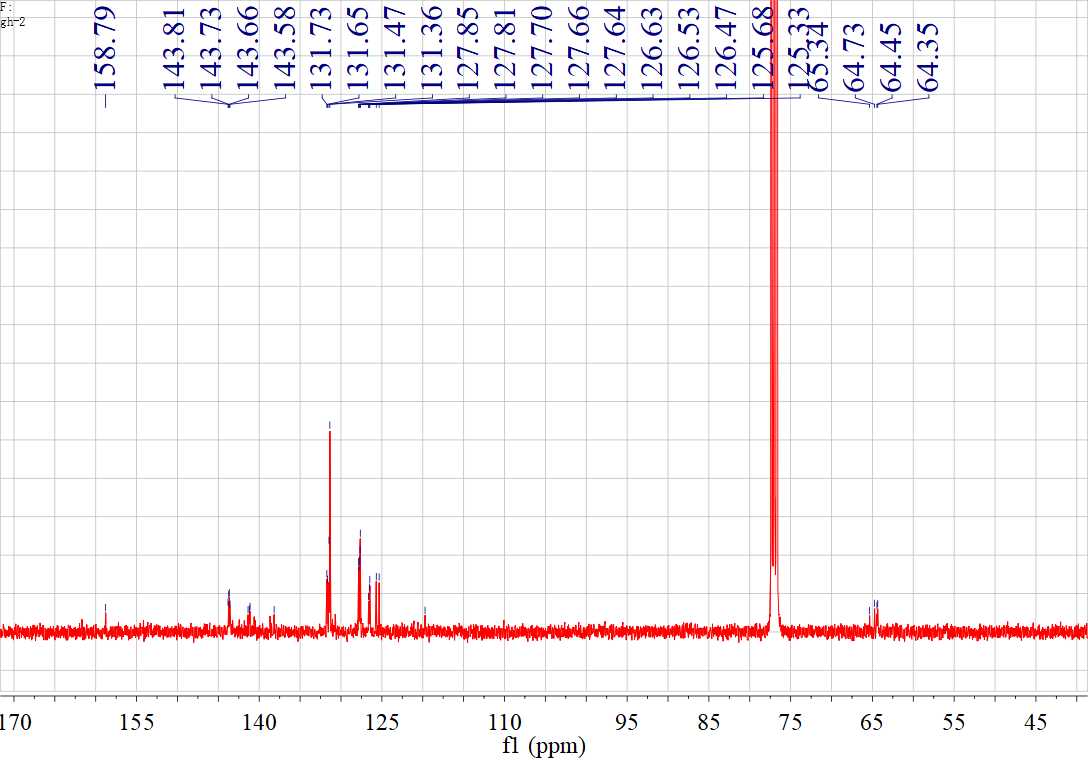


Figure S8 ^13^C NMR of PS in CDCl_3_-*d*_6_


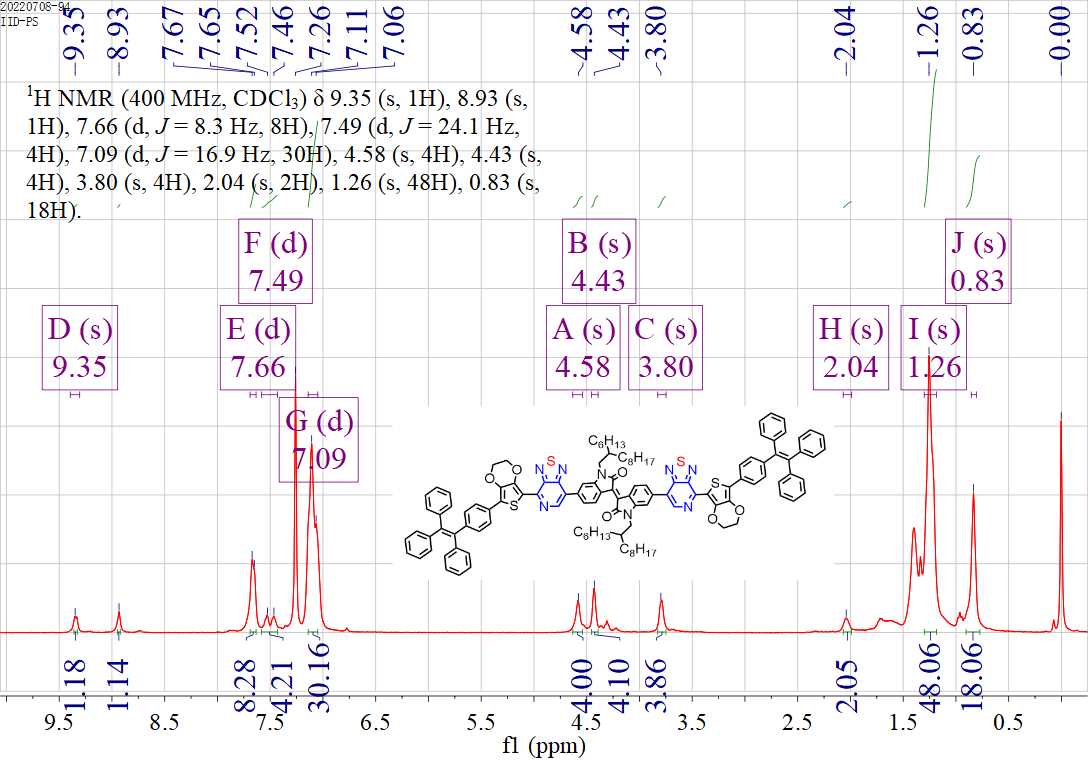


Figure S9 ^1^H NMR of IID-PS in CDCl_3_-*d*_6_


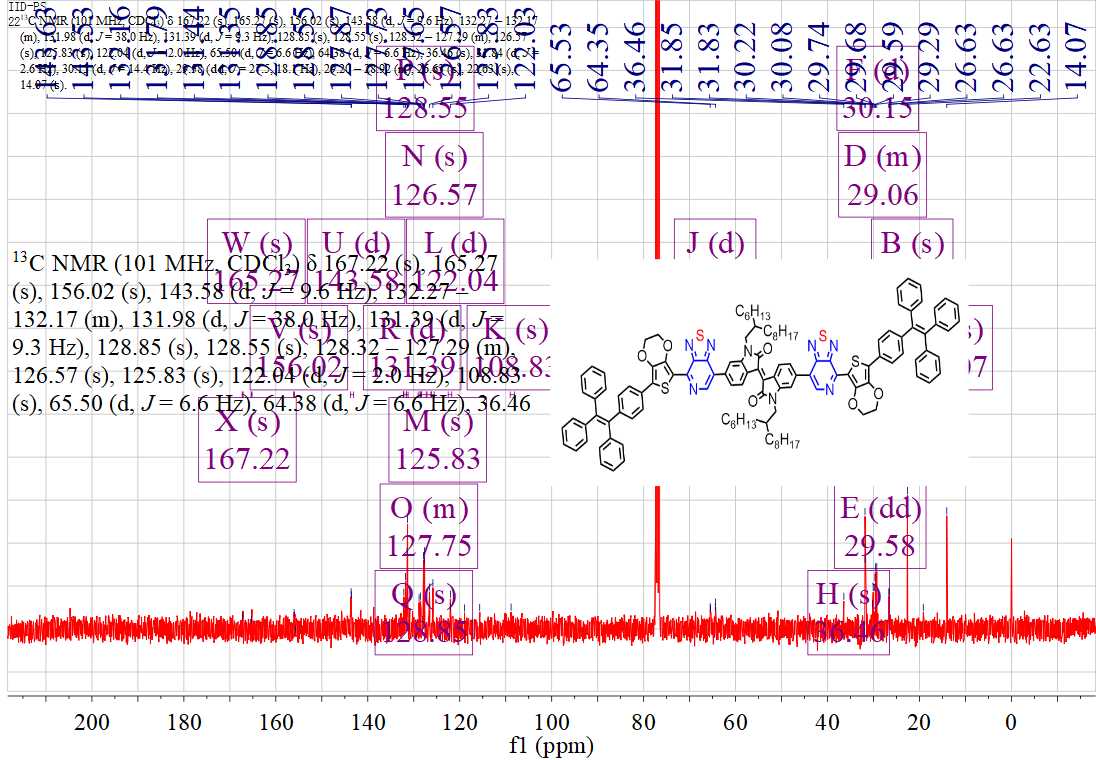


Figure S10 ^13^C NMR of IID-PS in CDCl_3_-*d*_6_


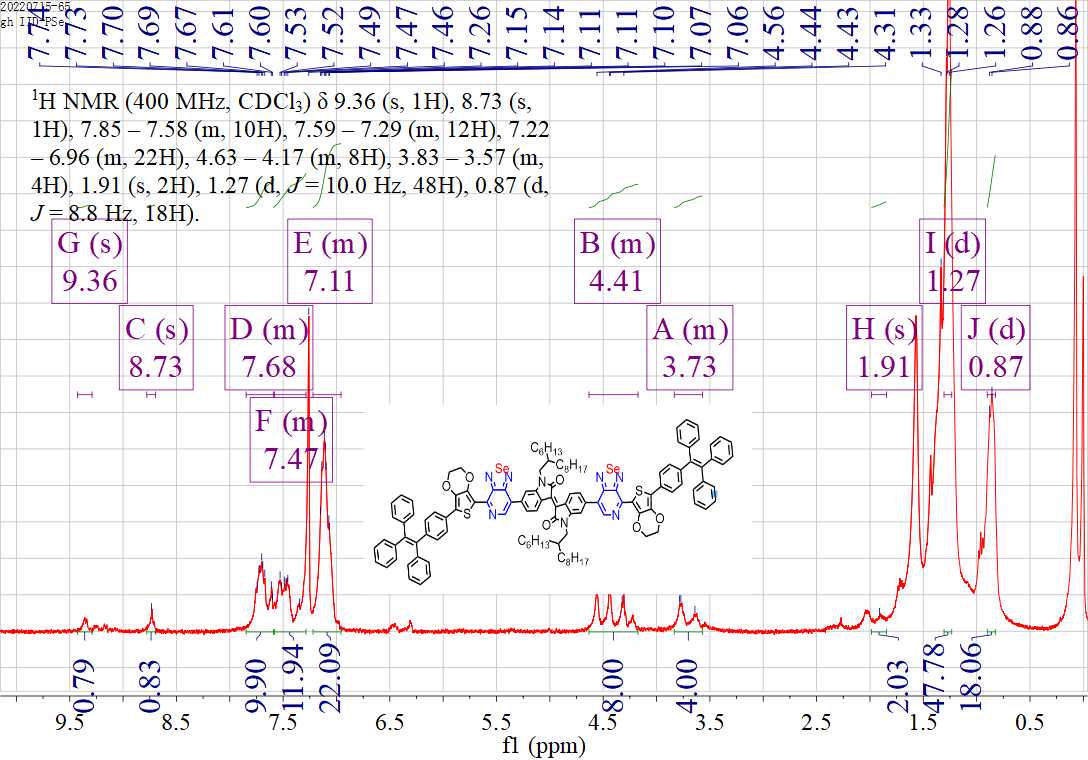


Figure S11 ^1^H NMR of IID-Pse in CDCl_3_-*d*_6_


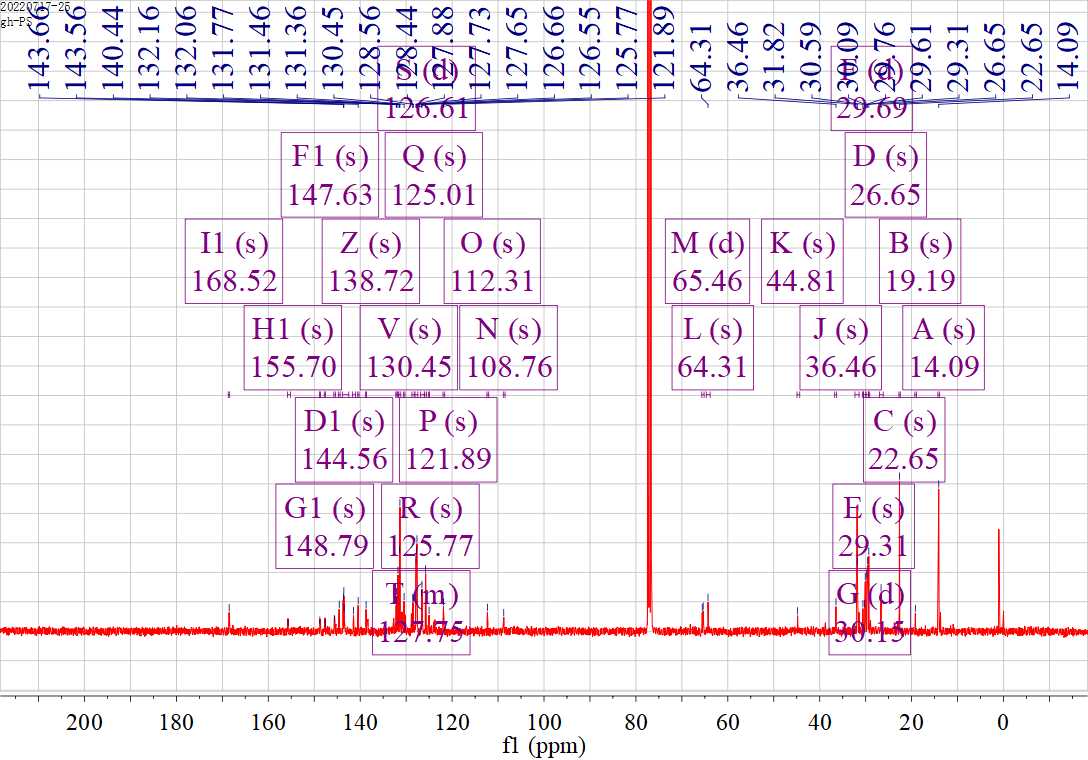


Figure S12 ^13^C NMR of IID-PSe in CDCl_3_-*d*_6_


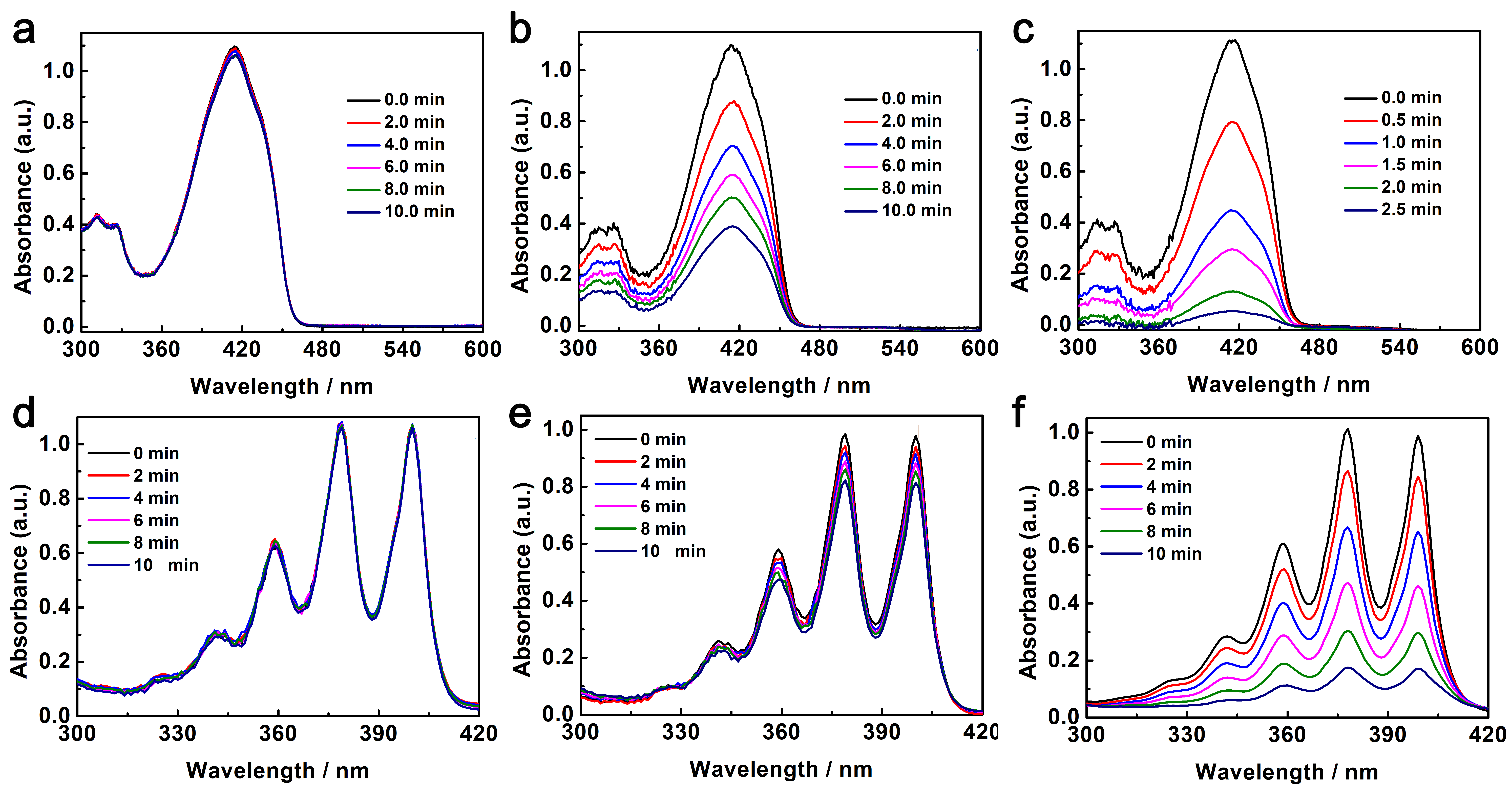


Figure S13 Study on photosensitization properties of phototheranostic moleculars and their nanoparticles. Absorption spectra of (a) DPBF, (b) DPBF + IID-PS and (c) DPBF + IID-PSe in CH_2_Cl_2_ with concentration of 25.0 μg mL^-1^, excitation wavelength of 671 nm and power of 15.0 mW cm^-2^; Absorption spectra of (d) ABDA, (e) ABDA + IID-PS NPs and (f) ABDA + IID-PSe NPs in PBS, with a concentration of 25.0 μg mL^-1^, excitation wavelength of 671 nm and power of 50.0 mW cm^-2^


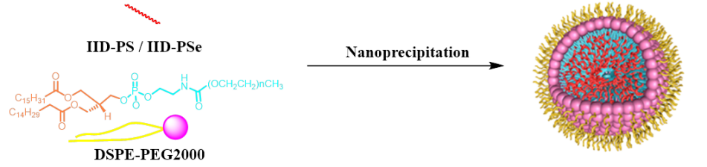


Figure S14 Schematic diagram of preparation of nanoparticles


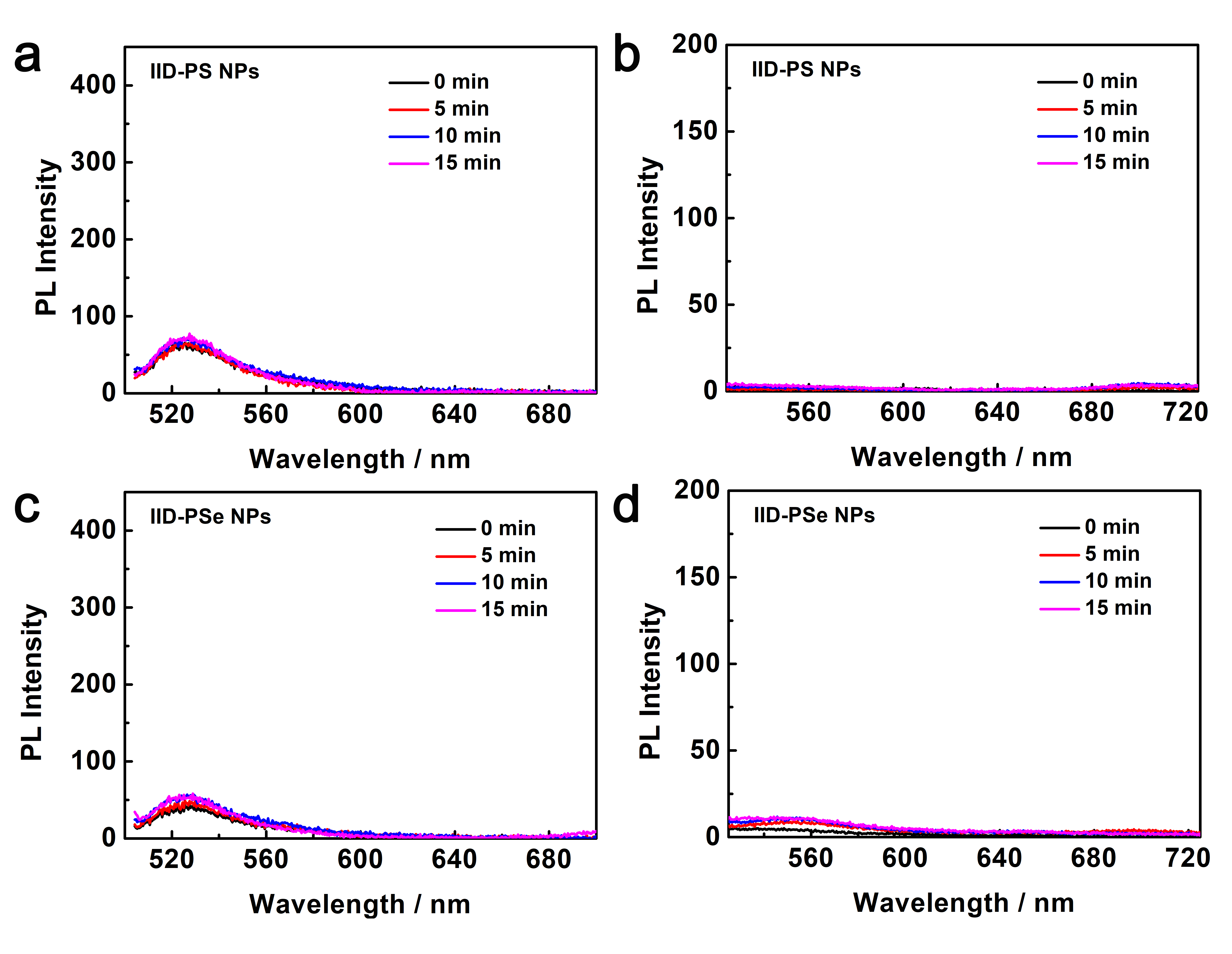


Figure S15 Reactive oxygen species detection with concentration of 25.0 μg mL^-1^, excitation wavelength of 671 nm and power of 50.0 mW cm^-2^. Superoxide anion Detection by DHR123 of (a) IID-PS NPs and (d) IID-PSe NPs (Ex/Em, 500/536nm). Hydroxyl radical detection by HPF of (b) IID-PS NPs and (e) IID-PSe NPs (Ex/Em, 490/515nm).


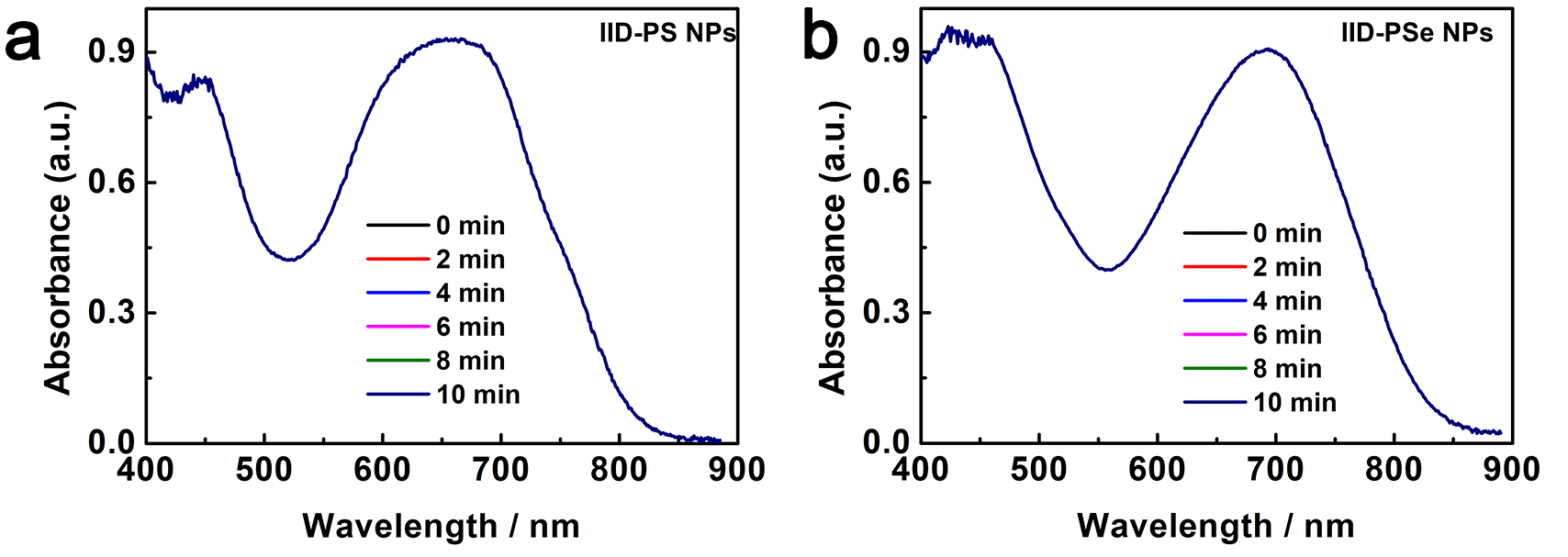


Figure S16 Photostability experiment of IID-PS NPs (a) and IID-PSe NPs (b) with a concentration of 50.0 μg mL^-1^, excitation wavelength of 671 nm and power of 50.0 mW cm^-2^


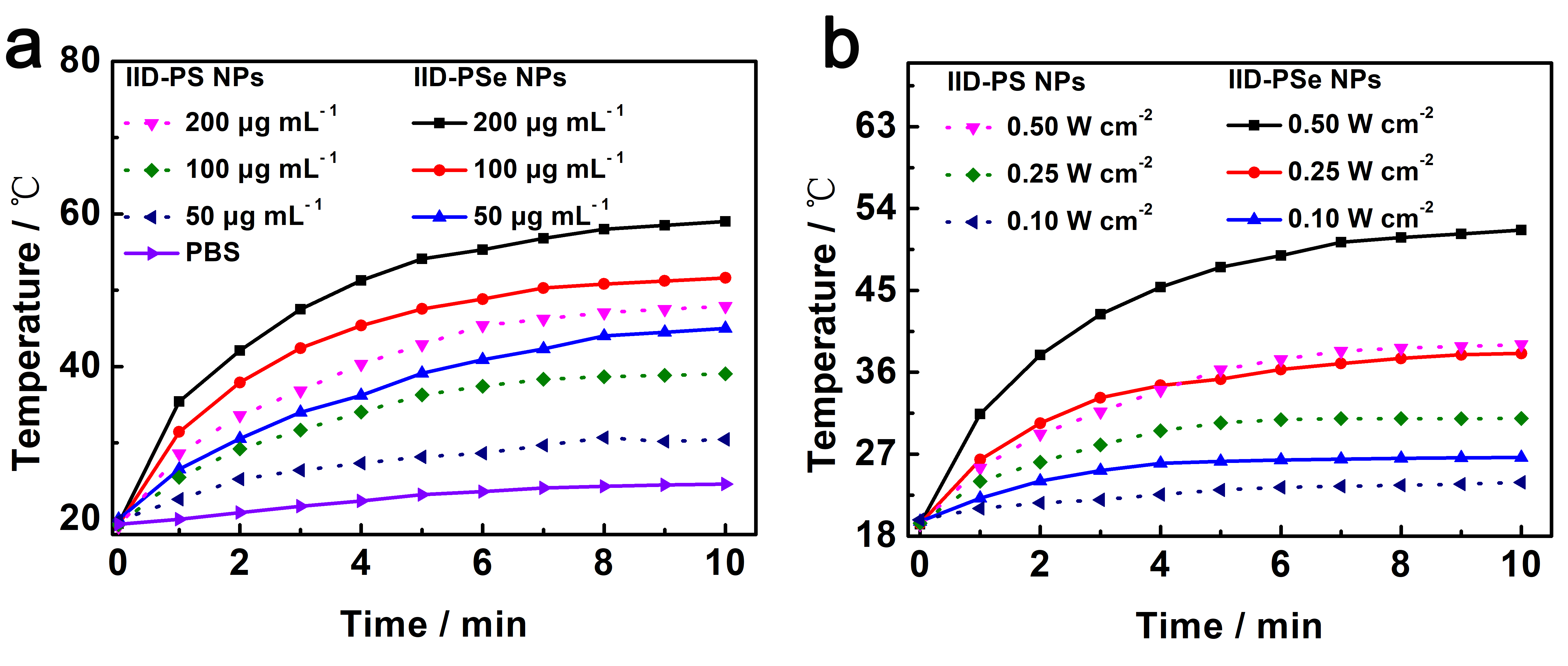


Figure S17 Study on photothermal properties of IID-PS NPs and IID-PSe NPs. (a) Dose-dependent photothermal heating curves of different concentrations nanoparticles under 671 nm laser irradiation (0.5 W cm^-2^); (b) different laser power-dependent photothermal heating curves of nanoparticles (100.0 μg mL^-1^) under 671 nm laser irradiation.


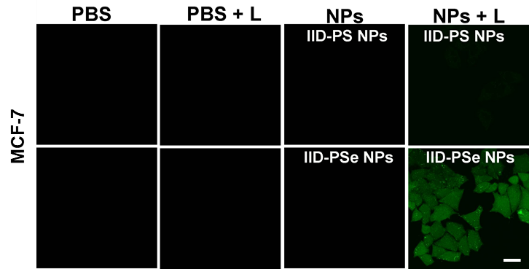


Figure S18 Intracellular Singlet Oxygen detection of MCF-7 cells with DCFH-DA as indicator in different treatment. Scale bar: 20.0µm; concentration: 25.0 μg mL^-1^; excitation: 671 nm; power: 50.0 mW cm^-2^; time: 10 min


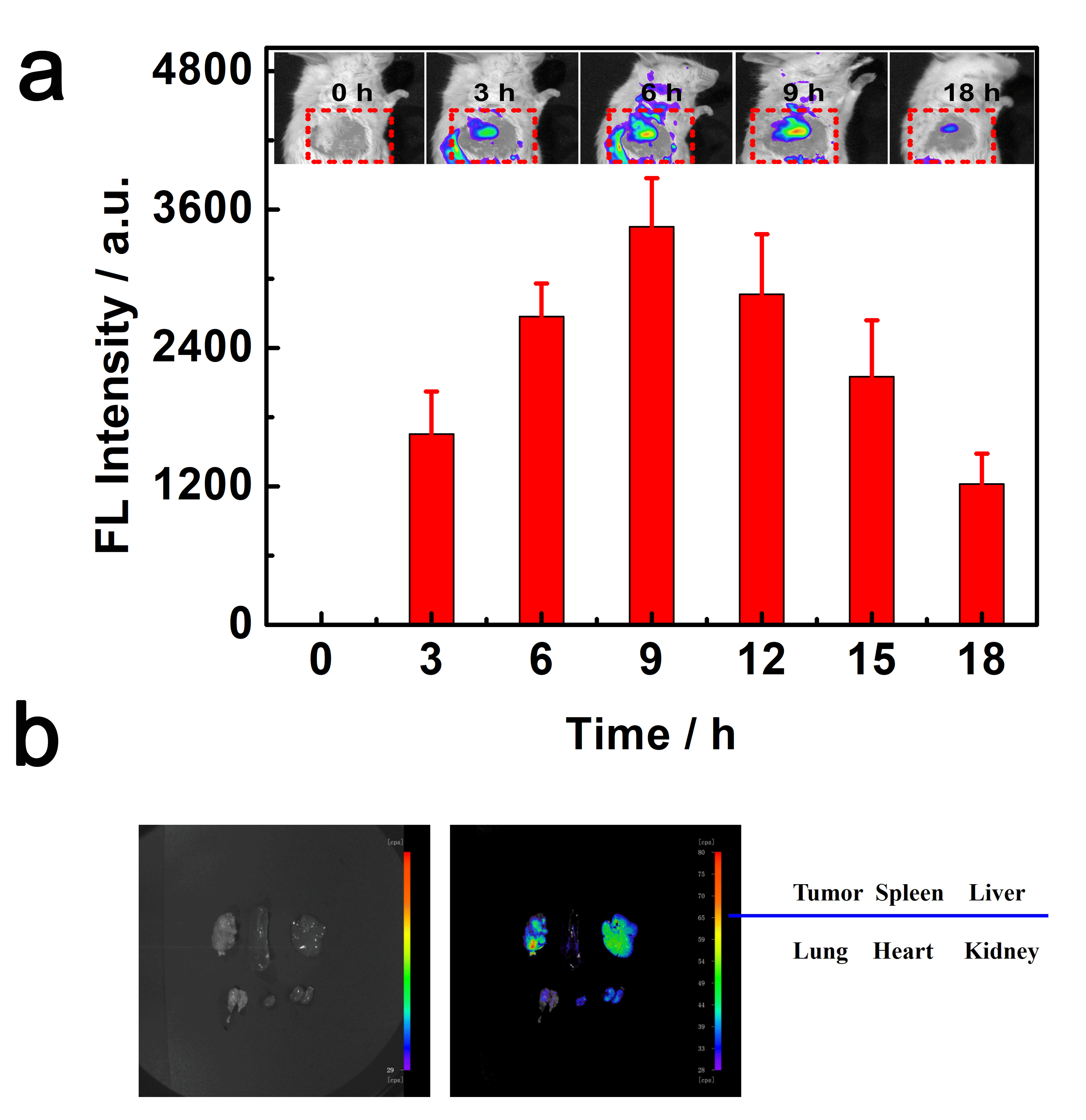


Figure S19 In vivo fluorescence imaging. (a) Tumor enrichment of IID-PSe NPs at different injection times in 4T1 tumor-bearing mice; (b) fluorescence imaging of major organs of mice after 9 h injection


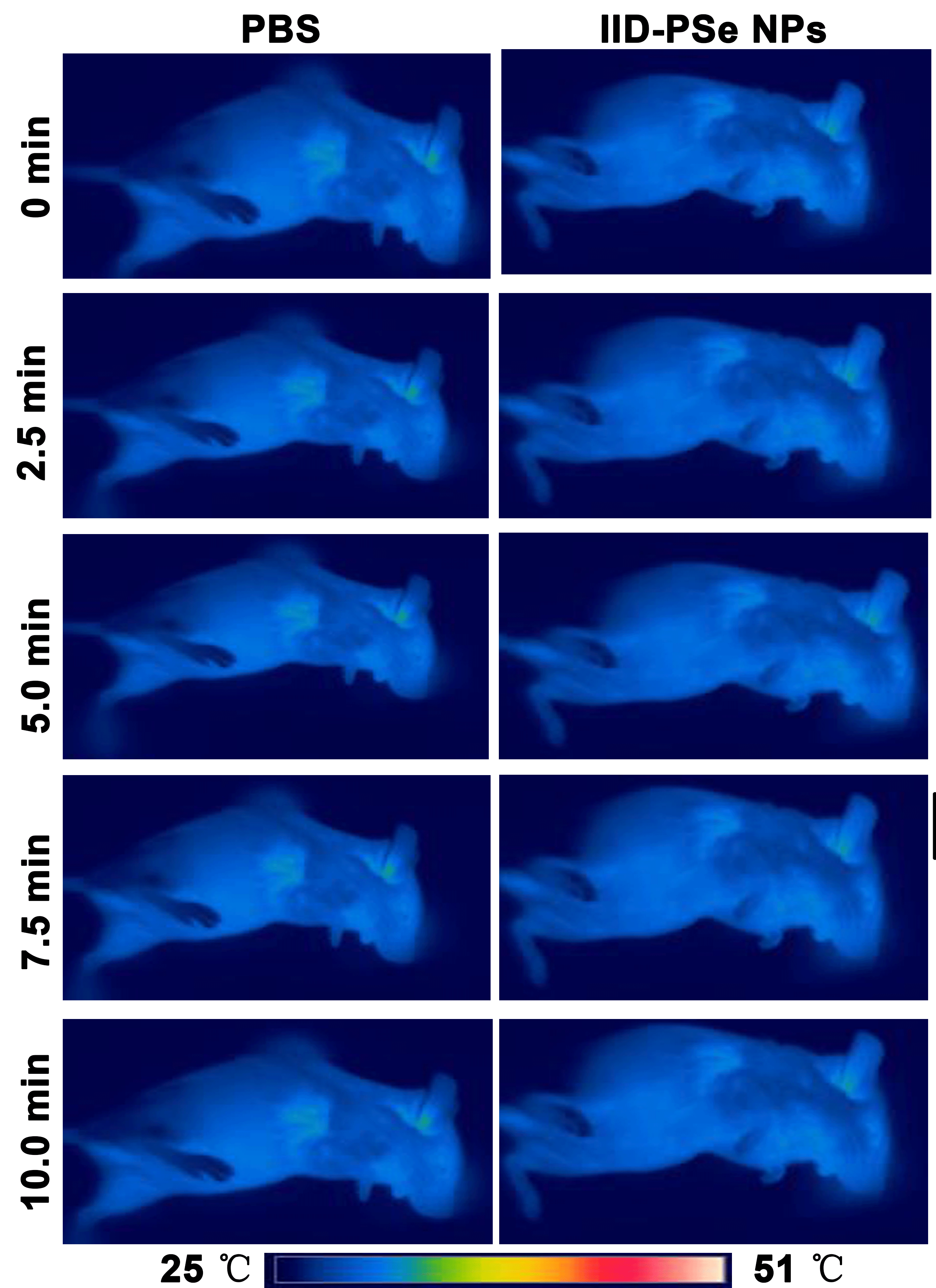


**Figure S20** (a) Photothermal images of 4T1-tumor-bearing nude mice after various treatments.


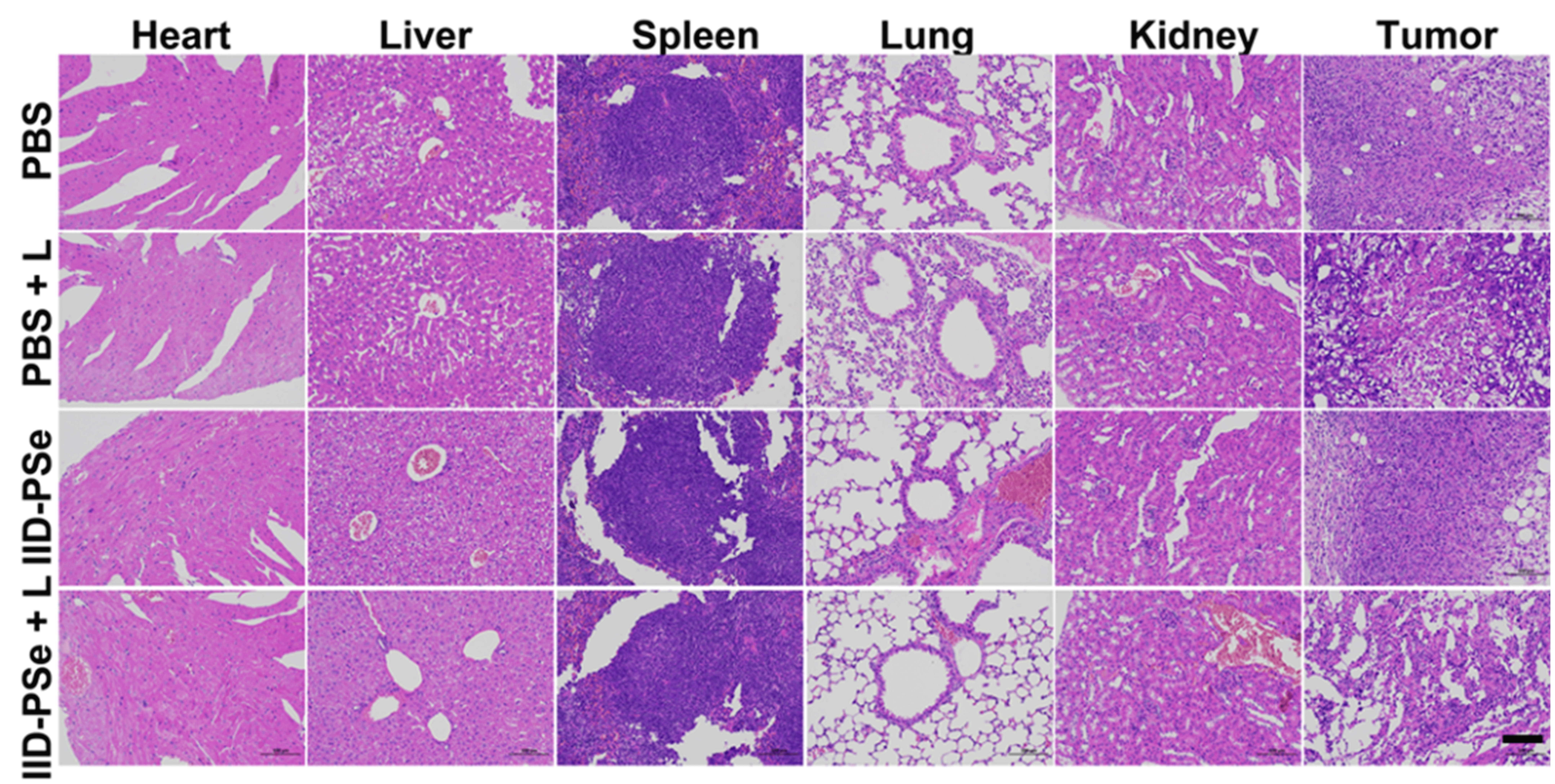


Figure S21 H&E staining of the major organs and tumors dissected from mice at 15 day after various treatments. Scale bar: 200 μm

Table S1 Test report of blood cells analyzer

| Parameters | without injection | 3th day post-injection | 6th day post-injection | Reference | unit |
| --- | --- | --- | --- | --- | --- |
| White blood cell | 1.5 | 5.2 | 1.5 | 0.8-6.8 | 10^9^ L^-1^ |
| Mononuclear cell | 0 | 0.1 | 0 | 0.0-0.3 | 10^9^ L^-1^ |
| Neutrophile granulocyte | 0.3 | 0.5 | 0.6 | 0.1-1.8 | 10^9^ L^-1^ |
| Leukomonocyte | 79.8 | 83.4 | 78.1 | 55.8-90.6 | % |
| Red blood cell | 8.19 | 8.02 | 7.79 | 6.36-9.42 | 10^12^ L^-1^ |
| Hemoglobin | 120 | 125 | 114 | 110-143 | g L^-1^ |
| Hematocritg | 39.8 | 38.7 | 37.9 | 34.6-44.6 | % |
| Mean corpuscular hemoglobin content | 14.6 | 15.5 | 14.6 | 15.8-19 | pg |
| Blood platelet | 464 | 838 | 503 | 450-1590 | 10^9^ L^-1^ |
